# Poly(beta-amino esters): applications in immunology

**DOI:** 10.1039/d5sc05951h

**Published:** 2025-12-10

**Authors:** Hulya Bayraktutan, Rafał J. Kopiasz, Amr Elsherbeny, Pratik Gurnani, Cameron Alexander

**Affiliations:** a Division of Molecular Therapeutics and Formulation, School of Pharmacy, University of Nottingham Boots Science Building Nottingham NG7 2RD UK cameron.alexander@nottingham.ac.uk; b Department of Pharmaceutical Biotechnology, Faculty of Pharmacy, Hacettepe University Ankara 06100 Turkiye; c Department of Chemical Engineering and Biotechnology, University of Cambridge Cambridge CB3 0AS UK; d Warsaw University of Technology, Faculty of Chemistry Noakowskiego 3 St. 00-664 Warsaw Poland; e Ex Vivo Cancer Pharmacology Centre, Translational Medical Sciences, Biodiscovery Institute, School of Medicine, University of Nottingham Nottingham NG7 2UH UK; f UCL School of Pharmacy, University College London 29-39 Brunswick Square, Bloomsbury London WC1N 1AX UK

## Abstract

The immune system is critical in safeguarding human health against a variety of external and internal threats, yet its dysregulation can lead to serious diseases such as autoimmune disorders and cancers. However, recent years have witnessed notable progress in immunotherapeutic interventions, including the development of novel modalities such as messenger RNA and DNA vaccines, immunotherapies using immune checkpoint inhibitors, and personalised dendritic cell vaccines. To optimise the delivery of these promising therapies, there is a growing interest in understanding how to target efficiently immune cells and immune-rich organs. Among the various delivery systems explored, synthetic polymers have emerged as versatile platforms, offering tuneable properties to modulate the immune system effectively. Poly(beta-amino esters) (PBAEs), characterised by their pH-dependent degradability and nanoparticle-forming capabilities, have gained significant attention as a promising delivery system in immunotherapy, immune-oncology, and vaccination. This review evaluates recent advances and applications of PBAEs in immunology. Beginning with an overview of synthetic methodologies, we cover their utilisation for nucleic acid, small molecule, and protein delivery, addressing therapeutic challenges associated with the immune system. We explore the diverse monomer chemistries employed in these applications and evaluate PBAEs in cutting-edge approaches such as mRNA and DNA vaccines, cancer immunotherapies, and immune cell reprogramming. By evaluating existing literature, this review aims to provide insights into the potential of PBAEs in immunotherapy while defining future directions for exploration in this rapidly evolving field.

## Abstract 2

Poly(beta-amino ester) (PBAE)s are versatile polymers which can be complexed with multiple nucleic acids to modulate immune responses.

## Graphical abstract

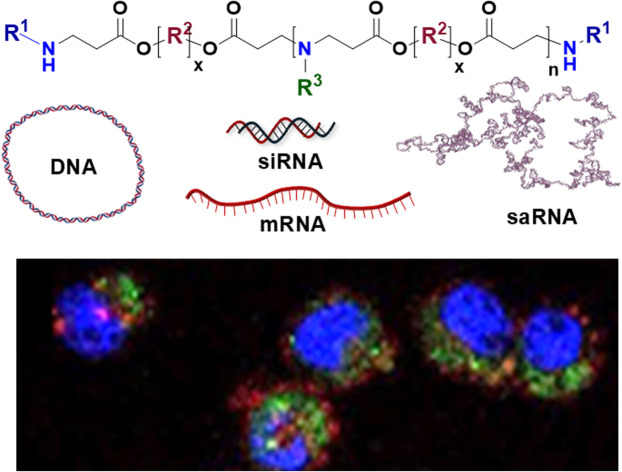

## Introduction

The immune system is central to many aspects of human health and disease. Its primary role is to detect, eliminate and protect from external pathogens (*e.g.* bacteria, parasites, fungi and viruses), environmental threats and endogenous diseases such as cancer.^1–4^ However, the immune system is also a key mediator of disease, including multiple autoimmune disorders (*e.g.* Addisons disease, Graves' disease, Hashimoto's thyroiditis, and rheumatoid arthritis) through an overactive or misplaced immune response, immune deficiency and cancers in immune cells (*e.g.* multiple myeloma, leukaemias and lymphomas) or their progenitors.^2,5^ Many of these can be severely debilitating, life limiting or cause death. Yet, the immune system can also be exploited for prophylaxis or therapy, with its role in vaccination widely utilised and increasing recognition that the immune system can be harnessed to eliminate classically untreatable disorders.^6,7^ The multifaceted role the immune system plays therefore opens the possibility for a spectrum of therapeutic targets, and a range of modalities to treat them directly or reprogram the immune system itself to treat or prevent disease.

In the last decade alone, we have seen breakthroughs, clinical trials and in some cases acceptance, of many critical immunotherapeutic or prophylactic modalities ranging from conventional small molecules, proteins, nucleic acid therapies and cell therapies. These include the first licensed messenger RNA and DNA vaccines, immunotherapies adjuvanted with immune checkpoint inhibitors, personalised dendritic cell vaccines and genetically engineered immune cells such as chimeric antigen receptor – T cells, with many of these revolutionising the outcomes for patients.^8–12^

To minimise negative effects and bolster efficacy of these new therapeutic modalities, understanding how to deliver them efficiently to immune cells and immune rich organs has become of significant importance.^13,14^ To date. Researchers have investigated many classes of delivery system, ranging from lipid-based particles, inorganic materials, electrospun fibres and hydrogels to name a few, which can be administered through injection, implantation, inhalation and more.^15–19^ Among these delivery approaches, polymeric materials have also emerged as a highly functional delivery platform, which can be tuned to target or directly modulate the immune system for therapy.^20–24^ Synthetic polymers are ideal for such applications as their versatile chemistry (molar mass, architecture, monomer composition, monomer sequence) enables them to be tailored for specific applications.^20–22,25^ Furthermore, the ability to impart biodegradability, and responsiveness to endogenous stimuli make them advantageous for biomedical applications. Indeed, there are several reviews which highlight key aspects of synthetic polymeric materials in immune related applications, with notable examples including immune instructive and targeting glycosylated synthetic polymers, delivery of immunostimulatory compounds through polymer drug conjugates and adjuvants for vaccines.^21^

This review focuses on recent advances in poly(beta-amino ester)s (PBAEs) a class of synthetic polymer, containing repeating tertiary amines, which have been considered as promising delivery systems in immunotherapeutic, immune-oncology and vaccination applications. PBAEs have numerous advantages as a drug delivery system, including their intrinsic pH-dependent degradability through chemical hydrolysis and excellent ability to form nanoparticles with negatively charged molecules.^26–29^ Initially synthesised *via* a Michael addition-based step-growth polymerisation by Chiellini in 1983,^30^ based on the seminal work on the analogous poly(amidoamines) by Ferruti in 1970,^31^ their popularity in the following years has spurred from their excellent performance as non-viral vectors for genetic material due to their polycationic nature, first demonstrated by Langer *et al.*^32,33^ Since these pivotal findings, hundreds of publications now report PBAEs as delivery systems not just for nucleic acids, but numerous biologically important macromolecules and chemotherapeutics, with many focusing on immune related applications.^28,34–37^ Here, we review and critically analyse the literature of PBAEs developed for, and used in, immunology applications. We first cover the basic synthetic methodology for producing PBAEs, followed by a comprehensive review of PBAEs used for nucleic acid (including DNA, mRNA, oligonucleotides and nucleic acid adjuvants), small molecule and cell delivery, which have tackled a therapeutic challenge associated with the immune system. We discuss aspects related to the monomer chemistries utilised for these applications and evaluate PBAE use in advanced applications, including mRNA and DNA vaccines, cancer immunotherapies and immune cell reprogramming approaches. We believe this is the first review on this topic hence have covered all the existing literature we could identify. We selected studies which employed PBAEs either for a direct application in the immune system, or fundamental studies to optimise delivery to immune cells. Finally, we conclude by discussing future considerations for PBAEs in immunology applications and avenues for further exploration.

## Synthesis and tunability of PBAEs

**Fig. 1:**
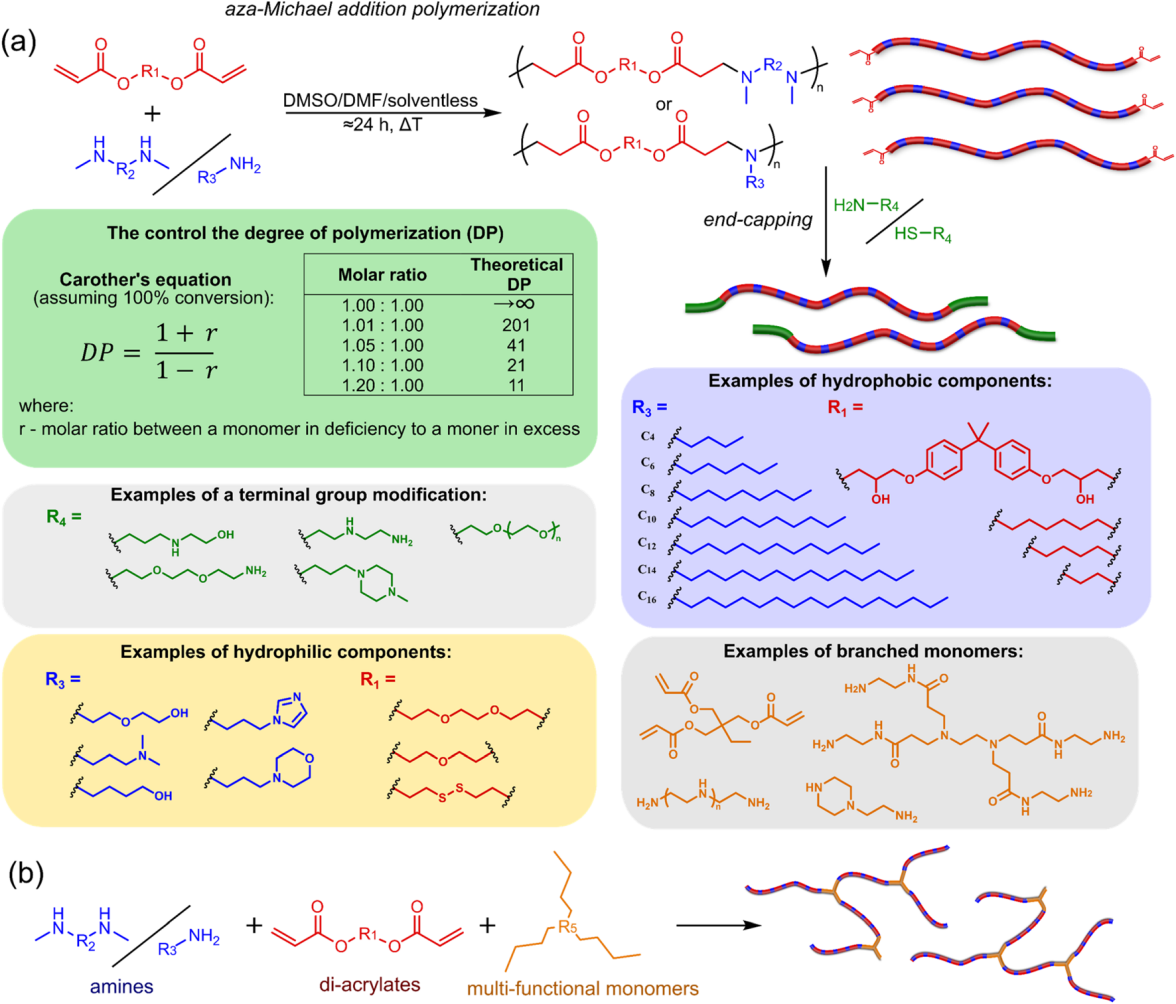
(a) The most popular routes for PBAE synthesis through aza-Michael polyaddition reaction together with the subsequent end-group modification *via* further aza-Michael or thia-Michael reactions. Examples of widely used functional groups used to tailor PBAEs properties for specific drug delivery applications. The Carother's equation allows estimation of DP of the final polymeric product in step-growth polymerisation. (b) Synthesis of branched PBAEs and examples of the most exploited multifunctional monomers.

The most popular way to synthesise PBAEs is aza-Michael addition polymerisation between amines and diacrylate esters (Fig. 1a). This involves a very simple synthetic protocol, and the reaction does not lead to many side products, so the final product does not require extensive purification and may be used directly for biological assays.^26^ Another advantage of aza-Michael addition polymerisation is the vast availability of commercial monomers as different di-, tri- and tetra-acrylates as well as amines. Therefore, this highly atom-efficient polymerisation allows researchers to produce easily a large library of PBAEs and to optimise for delivery of a desired cargo for a specified application. Since the first reports, many hundreds of PBAEs have been synthesised and tested as delivery vectors for nucleic acids, which have been summarized in numerous review papers.^28,34,38–42^ Beside nucleic acid delivery, many other macromolecules and small drugs have been successfully delivered by PBAEs.^35,43–45^

Aza-Michael addition, the mechanism behind PBAE synthesis, obeys third order kinetics (second order in amines and primary in acrylates) and occurs very rapidly even at room temperature.^46,47^ However, as polymer synthesis requires nearly quantitative conversion of monomer reactive groups, polymerisation is usually conducted at elevated temperature (40–110 °C) to shorten the reaction time to around 12–24 h. Importantly, as aza-Michael addition polymerisation is third order, its rate is strongly dependent on substrate concentration and so a high concentration of monomers is required to ensure the desired conversion to polymer within a reasonable time. Another advantage of a high monomer concentration is a reduction in the side product content. All step growth polymerisations, including aza-Michael addition polymerisation suffer from a formation of undesired macrocyclic compounds,^48–50^ and the contribution of macrocyclization reaction over polymerisation is promoted at low monomer concentration.^51,52^ Usually, these macrocycles may be easily detected in size-exclusion chromatography (SEC) traces as very narrow peaks at low molecular weight, and the cyclics can be removed *via* dialysis.^53–55^ Unfortunately, this requires an additional purification step, therefore, it is more convenient to prevent macrocycle formation by applying as high concentration of monomer as possible. However, the high concentration of the growing polymer may lead to very high viscosity of the reaction mixture.

Another crucial parameter in planning PBAE synthesis through aza-Michael addition polymerisation is solvent selection. Primarily, all monomers and polymeric products should be fully soluble in the chosen solvent to assure a proper course of polymerisation. A precipitation of oligomers and short polymers may suppress formation of high molecular weight polymers, whereas low solubility of monomers requires a high solvent volume, thus low monomer concentration slowing down the reaction and increasing cyclic side product formation.^35,46^ The most popular solvents for aza-Michael addition polymerisation are CH_2_Cl_2_,^56,57^ THF,^32^ DMF,^58^ and DMSO.^59,60^ Alcohols are generally avoided due to hydrogen bond formation between solvent and amines, which lowers their reactivity toward acrylates slowing down aza-Michael addition. Considering the potential toxicity of the residual solvent in the final polymer, DMSO seems to be the best choice due to its relatively good safety profile. Additionally, the most common way of handling PBAEs is a preparation of a concentrated stock solution in DMSO, which is subsequently diluted in an appropriate buffer. Another popular approach is aza-Michael addition polymerisation without solvent, which is possible because most monomers are liquids.^61^ Such a methodology allows for the highest monomer concentration thus the highest reaction rate and the lowest fraction of cyclic by-products.

It has been shown that the type of terminal groups^62–64^ and the molecular weight of PBAEs^65,66^ have an enormous impact on the delivery efficacy and toxicity, therefore these parameters need to be carefully fine-tuned for a targeted application. In aza-Michael addition polymerisations, these may be easily controlled by a simple manipulation of monomer ratio, and the degree of polymerisation (DP) of the final product may be estimated using Carother's equation (Fig. 1a).^67,68^ However, one should keep in mind that this prediction assumes a quantitative monomer conversion into the polymer without side reactions. In general, the longest polymer may be obtained using monomers at the exact stoichiometric ratio, and an increasing excess of one monomer decreases the molecular weight of the product terminated with the monomer used in excess. The most popular way for end-group modification with both small molecules and other polymers (end-capping) is *via* aza-Michael addition reaction with excess of bis-acrylic monomers leading to polymers terminated with activated double bonds (Fig. 1a). These groups are then accessible for further functionalization *via* subsequent thia-Michael and azo-Michael additions.^69–71^ It is important to not leave uncapped acrylic end groups as these have been shown to limit nucleic acid delivery.^65,72^

The numerous commercially available primary amines and bis-acrylates allow us to explore a wide landscape of chemical functionalities of PBAEs and to tailor their structure for a specific application. For instance, one may synthesize a library of PBAEs characterized by a range of hydrophobicity, hydrophilicity (Fig. 1a), and an overall hydrophilic-lipophilic balance, to balance toxicity and efficacy. Usually, the introduction of strongly hydrophobic groups to polycations stabilizes particles but also increases their toxicity.^71,73–75^ Furthermore, functional monomers containing different bases like tertiary amines, imidazole, or morpholine may be used to fine-tune p*K*_a_ of PBAEs, thus their behaviour at low pH.^76^ For example, it has been shown that carrier p*K*_a_ values play a key role in nucleic acid delivery, potentially due to the proton-sponge effect after transit of the polymer/nucleic acid complexes to endosomal compartments, and the influx of protons during the endosomal maturation process which may enhance escape of the complexes into the cytosol.^77,78^ Another broadly applied functionality in the PBAE family of materials is the disulfide bond, which can be installed in the polymer main-chain, and which is reported to be cleaved intracellularly by high cytosolic concentrations of glutathione.^79–81^ In turn this results in the breakdown of the PBAE into smaller fragments, which bind nucleic acids less effectively, resulting in enhanced cargo release intracellularly.^82–85^ To further explore the chemical structures of PBAEs, the degree of branching may be tailored by tuning the ratio between bis-functional monomers and multi-functional ones (Fig. 1b),^86–89^ which may lead also to hydrogel formation.^90,91^

### Intrinsic immunogenicity of PBAEs

The majority of this review covers PBAEs as delivery systems for therapeutic cargos (*e.g.* nucleic acids and proteins) which can be used for immunology related applications.^111,112^ However, the intrinsic immunogenic properties of PBAEs should also not be ignored. Polycations are now known to interact strongly with cell membranes stimulating a downstream pro-inflammatory cascade, which could therefore have unique adjuvant properties, especially when combined with a therapeutic modality.^113–115^

To this end, Jewell and co-workers have reported three elegant studies examining the intrinsic immunogenic properties of PBAEs, evaluating the mechanism and relationships with PBAE structure.^103,116,117^ Their first study aimed to identify the role of polymer degradation in the intrinsic immunogenic properties of PBAEs.^116^ This is critically important because PBAE delivery systems are likely to undergo degradation into smaller fragments which may attenuate the biological response of the original PBAE. They found that PBAE particles, produced from a PBAE based on 1,4-butanediol diacrylate and 4,4′-trimethylenedipiperidine (Table 1, entry 12), formed through electrostatic interaction with a SIINFEKL peptide antigen to mimic common vaccine approaches, strongly activated dendritic cells, promoted antigen presentation, and enhanced T cell proliferation. Interestingly, the immunostimulatory effects varied with polymer molecular weight, showing maximal stimulation at early degradation stages corresponding to high molecular weight, which diminished as degradation progressed. In contrast, free polymer exhibited no immunological activity. In mice, their PBAE particles increased the numbers and activation state of cells in lymph nodes. Mechanistic investigations revealed that this evolving immunogenicity correlated with changes in particle physicochemical properties and concentration during polymer degradation. This study highlights that the immunological profile of degradable synthetic polymers is highly dependent on molar mass/degradation, an important feature given the step growth polymerisation of PBAEs.

**Table 1:** Selected examples of PBAEs in immunology relevant application

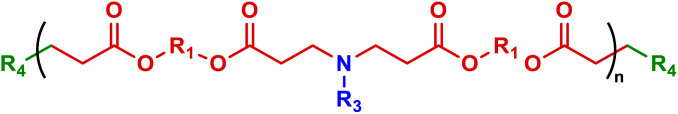							
No.	R1	R2/R3	R4	Cargo type	Target cells	Therapeutic target	Ref.
1^a^	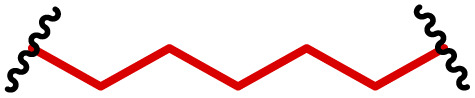	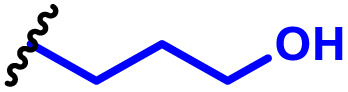	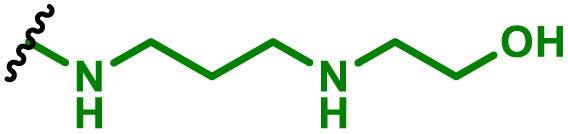	cDNA encoding sTRAIL protein	HepG2 cancer cells	Liver cancer – reprogramming of liver cells to locally secrete TRAIL	92
2^a^	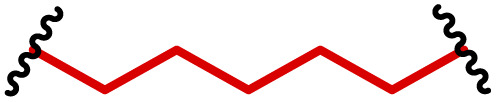	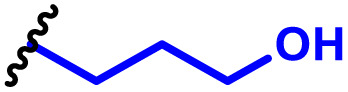	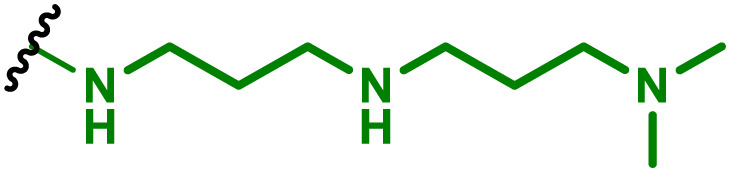	pDNA encoding 4-1BBL and interleukin-12	B16–F10 melanoma cells and xenograft tumor	Antitumoral – stimulation of T and NK cells	93
3^a^^,^^b^	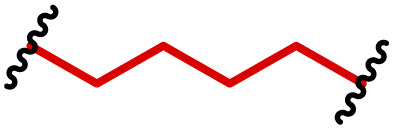	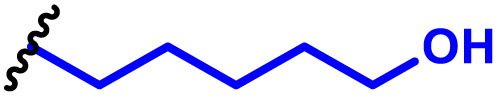	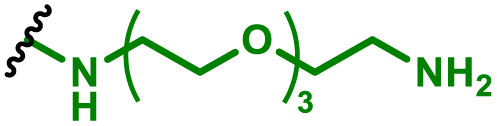	RP-182 peptide + DNA encoding ErbB2-specific CAR-T	Macrophages	Brain tumor – reprogramming of M2-like macrophages to M1-like phenotype	94
4^a^^,^^b^	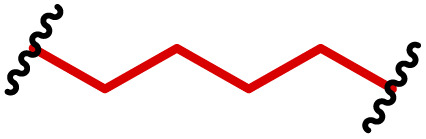	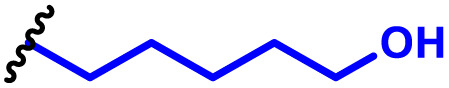	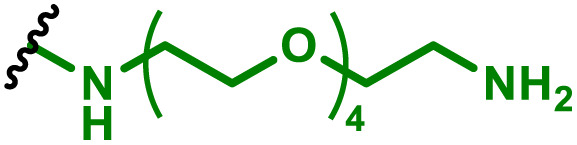	mRNA encoding OVA and TRP2	Dendritic cells	Vaccination – reprogramming cells to present specific epitope (OVA, TRP2) to be recognised by T cells	95
5^a^	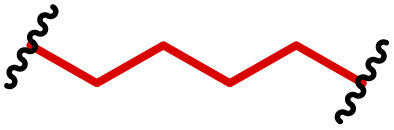	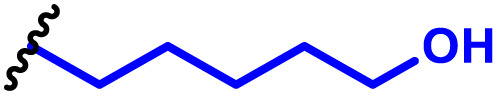	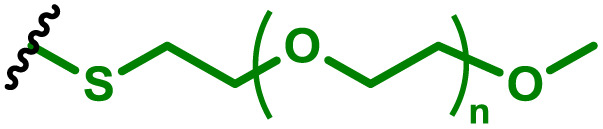	pDNA encoding the spike and the nucleocapsid of SARS-CoV-2	Dendritic cells (DC2.4)	Vaccination – reprogramming cells to present SARS-CoV-2 epitopes	96
6^a^	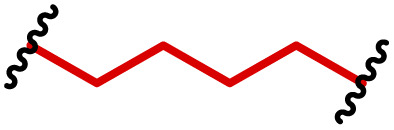	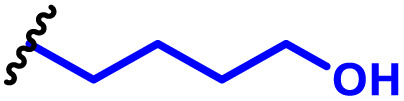	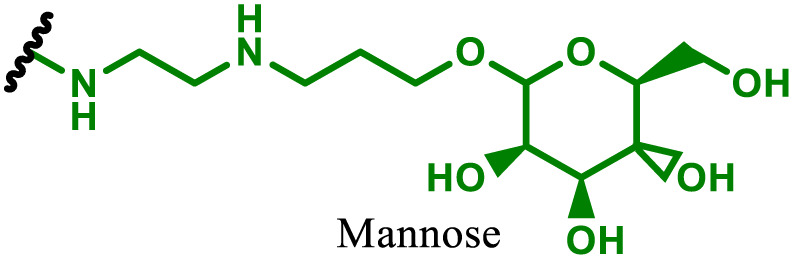	pDNA encoding OVA	RAW264.7, macrophages	Vaccination – reprogramming cells to present specific epitope (OVA)	97 and 98
7^a^	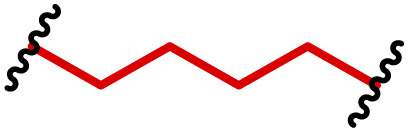	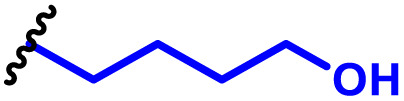	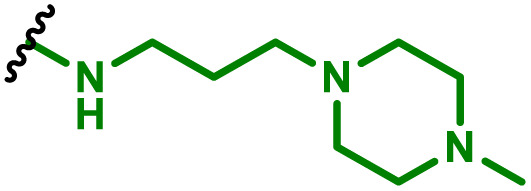	mRNA encoding M1-polarizing transcription factor TAMs	Ovarian cancer, melanoma, glioblastoma, macrophages	Cancer cells – reprogramming of M2-like macrophages to M1-like phenotype	4
8^a^	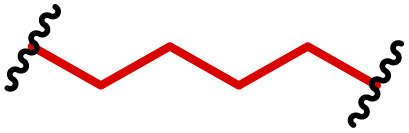	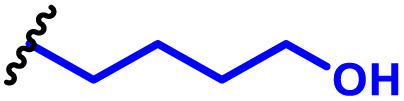	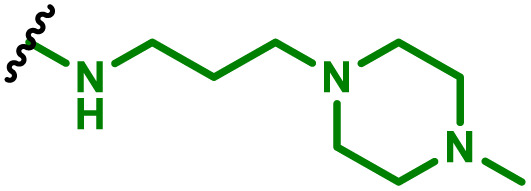	Cyclic dinucleotides (CDNs)	B16 melanoma tumors	Cancer cells – stimulation of the cytosolic (STING) signaling pathway	99
9	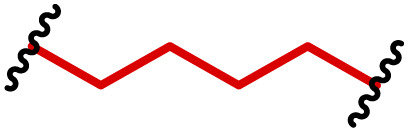	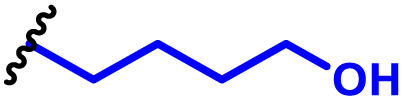	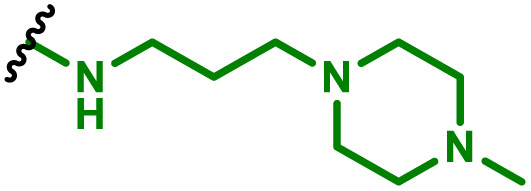	DNA encoding CAR + targeting antibody	T-cells	Introducing of leukemia-targeting CAR genes into T-cell	100
10	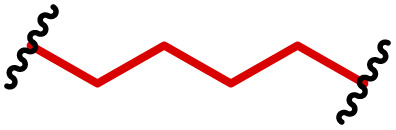	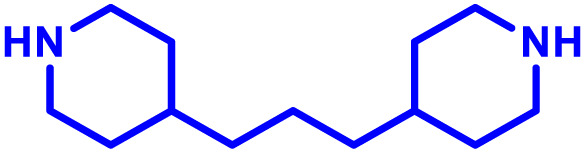	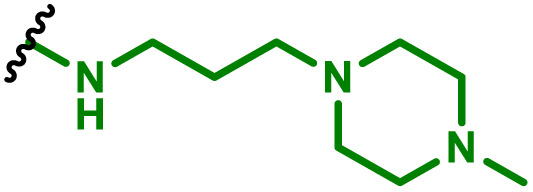	Antigens MHC's (KbIg-SI, DbIg-gp100 and KbIg-TRp2) and anti-CD28	T-cells	Antitumor – T-cell activation as an artificial APCs	101
11^a^^,^^b^	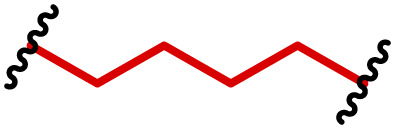	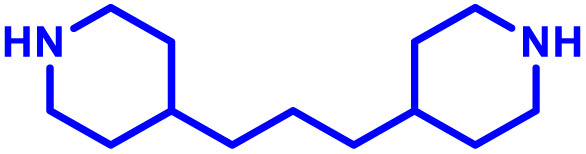	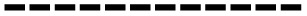	Reporter mRNA encodin luciferase	Dendritic cells (DC2.4)	Proof-of-concept for delivery to CD2.4	102
12	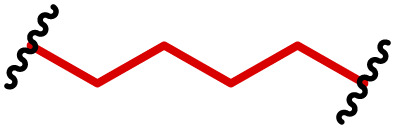	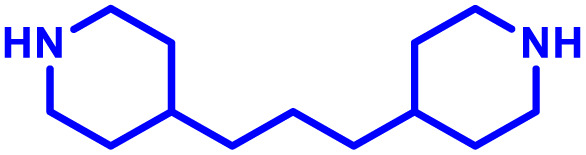	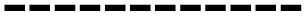	Control dicer-substrate siRNA + immunostimulatory effect of PBAE itself	RAW264.7 and RAW-Dual, macrophage cells	Activation of TLR and NF-ΚB pathway	103
13	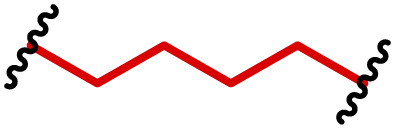	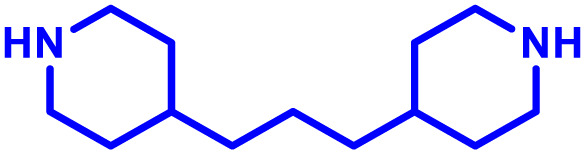	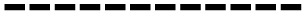	CpG oligonucleotide (CpG-ODNA)	Macrophages	Antitumor – activation of TLR9	104
14^a^	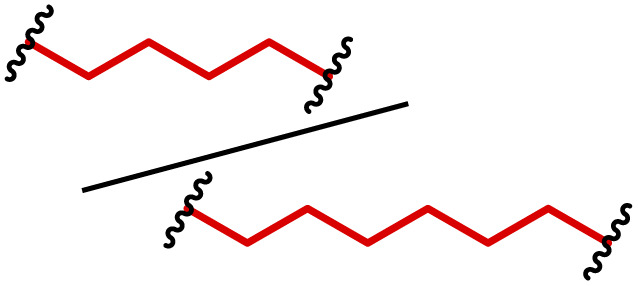	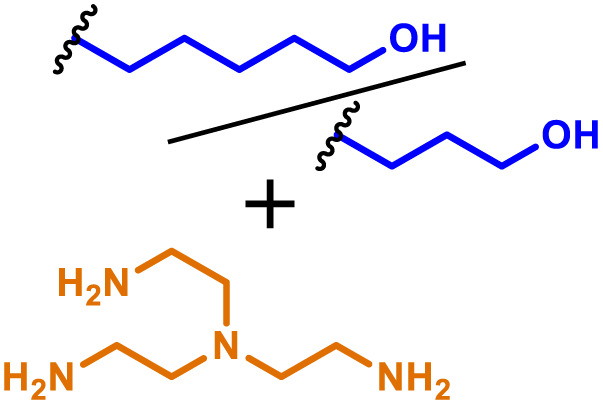	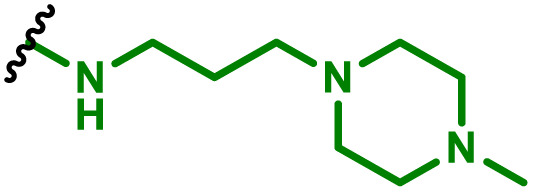	mRNA encoding a reporter protein (eGFP) + immunostimulatory effect of PBAE itself	Mouse bone marrow-derived dendritic cells (BMDCs)	Activation of antigen-specific immune response by PBAE itself	105
15^a^^,^^b^	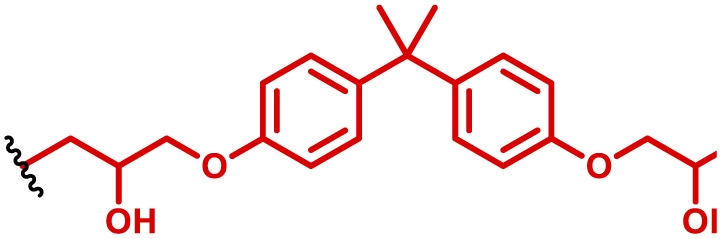	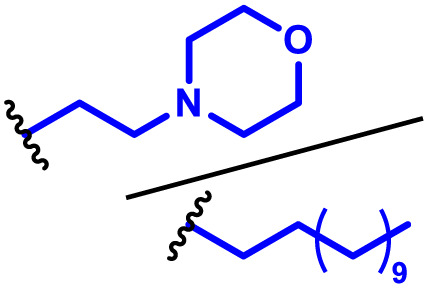	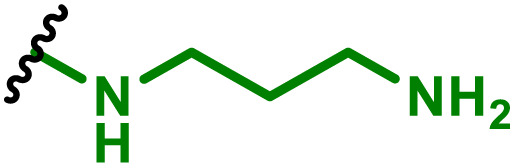	Reporting mRNA encoding Cre	Lung endothelium	Proof-of-concept for lung delivery	106–108
16^a^^,^^b^			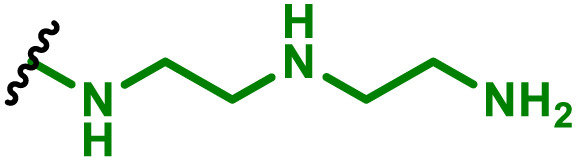	saRNA encoding rabies virus glycoprotein	Screening on C2C12 murine myoblasts; muscles (intramuscular injection)	saRNA encoding rabies virus glycoprotein; hybrid nanoparticles with lipids	109
17	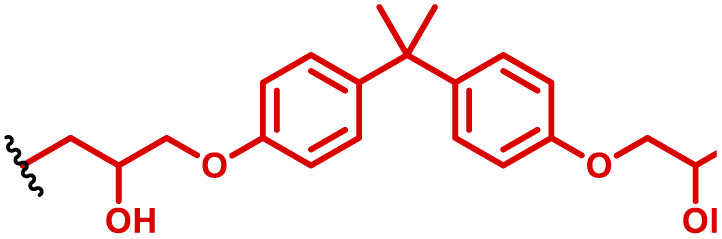	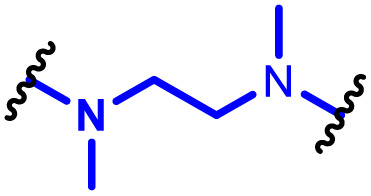	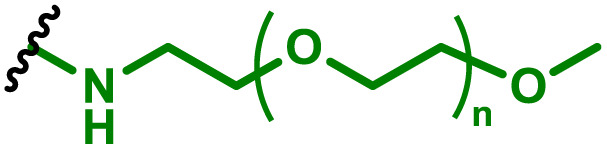	Poly(I:C)	Macrophages	Antitumoral – activation of TLR3 and MDA-5 receptors	110

a *In vivo* efficacy has been demonstrated.

b PBAE–lipid hybrid nanoparticle.

**Fig. 2:**
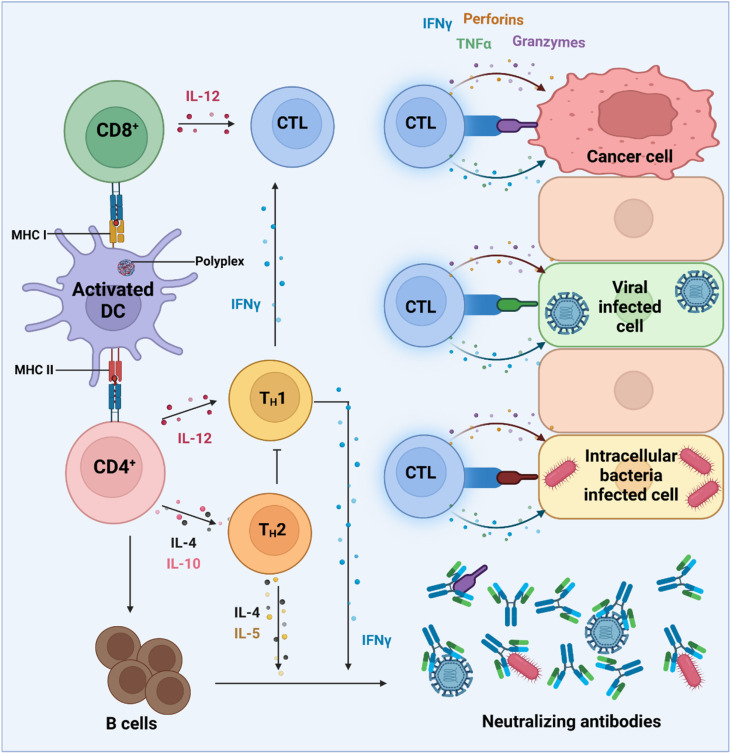
Activation of CD8^+^ and CD4^+^ T cells by antigen-presenting dendritic cells triggers distinct immune responses. Dendritic cells expressing antigens on MHC class I molecules activate CD8^+^ T cells, leading to their differentiation into cytotoxic T lymphocytes (CTLs) through interleukin signaling. CTLs, in turn, target and kill virally infected, cancerous, or intracellular bacteria-harboring cells. Meanwhile, dendritic cells presenting antigens on MHC class II molecules activate CD4^+^ T cells, resulting in the formation of TH1 and TH2 cells through distinct interleukin pathways. TH1 cells produce interferon-gamma, further enhancing CTL activation, while both TH1 and TH2 cells stimulate B cells to produce antibodies, augmenting the immune response against targeted cells.

The effect of molecular size on immunogenic properties of PBAEs was further investigated in a follow up study where a small range of PBAEs with varying starting molar masses but comparable and rapid degradation rates was synthesised.^117^ Treatment of primary DCs with free PBAEs, whether intact or degraded into low molar mass fragments, did not induce activation. Conversely, particles formed from PBAEs at various stages of degradation elicited distinct expression patterns of classical DC activation markers (*e.g.*, CD40, CD80, CD86, MHCII) and antigen presentation. Throughout degradation, the levels of activation varied alongside changes in physicochemical properties (*e.g.*, MW, concentration, size, charge). Notably, regardless of the initial molar mass, immunogenicity peaked when the MW of degrading PBAEs decreased to 1500–3000 Da (Fig. 2).

The mechanism behind these findings was then investigated, using PBAEs either in soluble form or complexed with an siRNA (DsiRNA) which does not bind to any part of the human or mouse transcriptome.^103^ It was found that PBAE complexes were able to stimulate expression of CD40, CD80, and CD86, however expression of proinflammatory cytokines IL-6 and IL-10 was not shown. Furthermore, investigations in TLR reporter cell lines, for TLR3, TLR7 and TLR9 indicated no activation of these pathways above the negative control using PBAE polymers or complexes with DsiRNA. Examination of the IRF and NF-kB pathways in RAW 264.7 macrophages revealed IRF activation using PBAE polyplex nanoparticles, which although are commonly downstream of TLR activation, seems to be independent of these. Although not directly identified in this study, it is clear that PBAEs can activate moderate immune responses in macrophage cells, but not in dendritic cells as shown in their previous work. Further studies could involve transcriptomic analysis to identify putative mechanisms.

### Delivery of nucleic acids

Nucleic acids exhibit many purposes for the propagation and function of life, most commonly for information storage *via* DNA in the genome, or as relays to produce functional proteins (though mRNA and tRNAs) from stored genetic information.^118^ Nucleic acids have been a critical target for medicine for decades, using small molecule drugs which can disrupt the structure or chemistry of DNA.^118,119^ These concepts have been the cornerstone for treatment of cancers and other diseases, including immune related malignancies. More recently, nucleic acids themselves have been recognised as a therapeutic modality. Examples include mRNA and plasmid DNA to instruct expression of a desired proteins or miRNA, siRNA and antisense oligonucleotides that can be employed to suppress expression of disease related proteins.^120–122^ These nucleic acid technologies have created the possibility for selective therapies which can reduce the off-target effects which can be observed with conventional small molecule therapeutics and which contribute to their side effects and poor tolerability.^123–125^

Within the context of the immune system, nucleic acids have been utilised in a few key prophylactic or therapeutic indications. Due to their ability to express any desired protein, antigens encoded by DNA and mRNA have been widely explored as tuneable vaccines against infectious disease or immunotherapies against cancer, whilst oligonucleotide therapies can be utilised as immune checkpoint blockade. The vulnerability of naked nucleic acids to degradation by endogenous nucleases and poor association with cell membranes necessitates the use of a delivery vehicle to transport these fragile molecules to their intracellular targets.^125^ The most popular of these have been lipid nanoparticles (LNPs) which are 50–200 nm self-assembled complexes composed of various lipid components.^126,127^ These operate by condensing nucleic acids into core–shell nanoparticles, protecting them, and enhancing translocation across the cell membrane. Despite LNPs being the most clinically advanced delivery approach, given their application in Onpattro (an LNP-siRNA therapy for hereditary transthyretin-mediated amyloidosis) and the mRNA COVID-19 vaccines, SpikeVax and Comirnaty, there is a need for alternative delivery approaches.^128^ Furthermore, LNPs current have a tight intellectual property space and may not possess the chemical diversity for all delivery challenges.

**Fig. 3:**
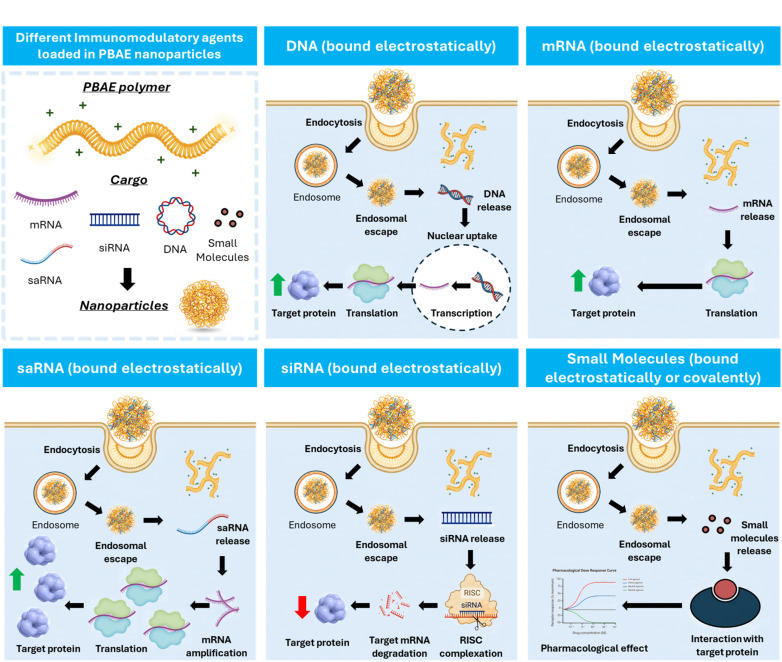
Schematic diagram illustrating the diverse cargoes carried by PBAE polyplexes (DNA, mRNA, saRNA, siRNA, small molecule drugs) and their intracellular fates: DNA polyplexes deliver DNA to the nucleus, where it is transcribed into mRNA by RNA polymerase and then translated in the ribosomes to produce the target protein; mRNA polyplexes release mRNA into the cytoplasm for direct translation in the ribosomes; saRNA polyplexes utilize a self-amplifying replicon to generate multiple mRNA copies, leading to increased protein production; siRNA polyplexes induce RNA interference through complexation with the RNA-induced silencing complex (RISC), silencing specific genes and reducing target protein production; small molecule drug-containing polyplexes target specific proteins/receptors to elicit pharmacological effects.

As mentioned above, PBAEs are highly versatile biodegradable polymer scaffolds, that can be controlled in terms of polymer backbone chemistry, pendant chain and end-groups, and which have been used in a variety of applications.^26,32,89^ The ionisable amine in the polymer backbone can be protonated at physiological pH or lower into polycations, enabling PBAEs to form electrostatic complexes with negative charged nucleic acids, sometimes called polyplex nanoparticles, as an alternative gene delivery approach to LNPs (Fig. 3).^42,129^ This was first achieved in 2000, when Lynn and Langer published their two pioneering studies combining a broad range of diacrylate and amine monomers to produce high throughput combinatorial libraries and enabling them to screen the effectiveness of PBAEs for *in vitro* DNA delivery efficacy.^32^ Following the foundation of PBAEs in the gene delivery field, hundreds of reports focusing on PBAEs as effective delivery systems for different nucleic acid cargos have now been published.^65,130^ As discussed previously, this adoption into the gene delivery scene is largely due to the ability to tune the materials chemistry and encode specified release nucleic acid release mechanisms which cannot easily be achieved for other polymer or lipid delivery approaches.

The progression of nucleic acid therapeutics towards immune applications, such as immunotherapies and vaccines, has therefore led to the investigation of many PBAEs in immune cell applications. In this section we discuss the state-of-the-art literature of PBAE design for nucleic acid immunology related applications.

### DNA

DNA therapies most commonly consist of circular plasmid DNA to induce expression of a specified protein. Depending on the DNA sequence, expression of the desired protein can be achieved either transiently for applications including protein replacement therapies, vaccines for infectious disease, or instead permanently to replace faulty genes in hereditary disorders and to train various immune cells (*e.g.* dendritic cell or T-cells) to eliminate cancers. Due to the necessity to deliver DNA to the nucleus, either episomally or for integration the genome, the most prevalent delivery approach has been through adeno-associated viral (AAV) vectors.^131,132^ However, packing limitations on the nucleic acid size in AAVs, difficulties surrounding large scale biological manufacture, and concerns around vector mediated reactogenicity has therefore shifted attention to non-viral delivery approaches, including PBAEs.^133–135^ In this sub-section we discuss advancements in PBAE design for DNA delivery to immune cells or for applications in immunology.

One of the key advantages of PBAEs is their tuneable chemistry, both in the pendant chain and end-groups. This enables researchers to be able to decorate the resulting DNA polyplex nanoparticles with the desired surface chemistries, which could enhance trafficking and localisation on to immune cells to improve delivery specificity. Pfeifer and co-workers^97^ used this approach to great effect by employing a two-step approach to introduce mannose residues at the PBAE end groups (Table 1, entry 6). They first generated a small library of acrylate terminated PBAEs using different alkyl aminoalcohols and aliphatic diacrylates. The acrylate end groups were then first transformed into amine functionality then further with allyl-α-d-mannopyranoside to enhance potential uptake in antigen presenting cells (APCs) and macrophages, which overexpress mannose binding receptors. *In vitro* gene delivery assays using DNA polyplexes derived from the mannose PBAEs revealed a significant improvement in uptake and transfection efficiency within RAW264.7 macrophage cells compared to non-mannosylated controls. Furthermore, *in vivo* immunisation studies utilising ovalbumin encoding pDNA vaccine, administered intraperitoneally or subcutaneously, demonstrated that mannosylated PBAEs elicited a robust, efficient, and safe humoral immune response without the need for adjuvants when compared to control antigens delivered through genetic or protein means. Encouragingly there were no signs of adverse reactions or mortality. For both mannosylated and non-mannosylated polyplexes the total IgG titers were notably higher than those from the OVA pDNA positive control 14 days post inoculation. By day 21, (7 days after the second dose), only the mannosylated polyplexes showed significantly elevated antibody titers compared to the pDNA control, highlighting the improvement in APCs targeting through PBAE design.

In a follow up study, the same group^98^ then systematically evaluated how PBAE molecular weight and relative mannose content influenced polyplex properties and DNA transfection efficiency, using the same strategy to generate mannose end-group modified PBAEs. Within the library, polymers with the lowest molecular weight and highest relative mannose content exhibited gene delivery transfection efficiencies comparable to, or surpassing those of, commercial controls (JET-PEI, Xfect and FuGENE HD) in RAW264.7 macrophages when treated with luciferase encoding plasmid DNA. In their library they identified that the most effective polymers formed the smallest polymer–plasmid DNA complexes (approximately 300 nm) with moderately positive zeta potentials (less than 10 mV). Despite a lack of further *in vivo* investigations, this study highlights the versatility in PBAE design and the opportunity this brings to elucidate structure function relationships.

Immunotherapies are fast becoming a critical tool to treat cancer, typically by stimulating a cytotoxic immune response against tumour associated antigens to selectively destroy malignant cells. However, for these therapies to be effective, it requires coordinated signalling for APCs to instruct CD8^+^ T-cells towards the correct target. The most common approaches are to extract APCs and manipulate them *ex vivo* such that they express the patient-specific antigen, which is a complex process and required prior knowledge on the antigen itself. Green and co-workers^93^ reported a novel ‘antigen-agnostic’ approach using PBAEs to deliver pDNA encoding for 4-1BBL and interleukin-12, a tumour necrosis factor ligand present on activated T-cells and an APC secreted cytokine respectively to B16–F10 melanoma cells and xenograft tumours. In principle, this should reprogram cancer cells and modify the immunocompetency of the tumour microenvironment *in situ*. They initially produced a library of PBAEs, similar to that from the studies described by Pfeifer and co-workers above, but with a range of aminated endcaps and assessed their ability to transfect B16–F10 melanoma cells *in vitro* (Table 1, entry 2). The highest performing PBAEs (those with transfection efficiencies above 80% and metabolic activity above 80%) commonly included 1,4-butanediol diacrylate copolymerised with 4-aminobutanol and a piperazine (named 4-4-7 and 4-4-27 in original study) and or primary amine end-group, or 1,5-pentanediol diacrylate copolymerised with 3-aminopentanol and a dimethylamine functional end group (named 5-3-49 in original study). The top 3 performing PBAEs also showed efficient reporter gene expression in equivalent xenografts, with 5-3-49 exhibiting 20-fold luminescence over the negative control, and hence this polymer was taken forward as the lead PBAE candidate. When complexed with the immunostimulatory pDNA constructs, the 5-3-49 PBAE polyplexes elicited a strong interferon-gamma response, used as a proxy to assess potential T-cell and NK cell activation. Further *in vivo* studies using this reprogramming PBAE polyplex platform led to a significant reduction in tumor growth rate, in B16–F10 melanoma and MC38 colorectal cancer xenograft in certain instances, complete tumor clearance, especially when co-administered with anti-PD-1 checkpoint blockade therapy. Tumour analyses confirmed that locally administered APC-reprogramming nanoparticles stimulated a cell-mediated cytotoxic immune response, assessed through IFN-gamma expression and upregulation of genes associated with cytotoxic immune response, highlighting the translational potential of PBAEs for immunotherapy applications.

Chimeric Antigen Receptors T-cells (CAR-T) have now gained clinical authorisation by the FDA for treatment of a range of blood cancers, including acute lymphoblastic leukaemia, multiple myeloma and several lymphomas. These therapies operate by reprogramming collected T-cells outside of the body to recognise a tumour associated antigen and then using those T-cells to eliminate malignant cells once reinfused. The complex process of collecting, purifying, and manipulating T-cells *ex vivo* remains a significant manufacturing challenge limiting the scalability of these therapies. Stephan and co-workers^100^ have reported the use of PBAE delivered DNA cargos to reprogram T-cells *in vivo* which could significantly improve the clinical application of this emerging therapeutic modality (Table 1, entry 9). To achieve this, they devised a novel strategy by conjugating a microtubule associated sequence (MTAS) and nuclear localisation signal peptide (NLS) on to the PBAE to facilitate efficient delivery to the nucleus once internalised by T-cells. Enhanced uptake within these cellular targets was ensured by coating the PBAE/DNA polyplexes with anionic poly(glutamic acid) coated with a CD3 T-cell targeting antibody, anti-CD3e f(ab')2. Using these nanoparticles, the authors observed rapid uptake and translocation to the cytosol, while MTAS and NLS signalling enhanced transfection of these cells by 8-fold. To assess potential for tumour eradication, the authors delivered PBAE/DNA complexes to reprogram T-cells to target leukaemia cells. Following injection, tumours were eradicated in 7 out of 10 mice, leading to a 58 days improvement in average survival rates.

**Fig. 4:**
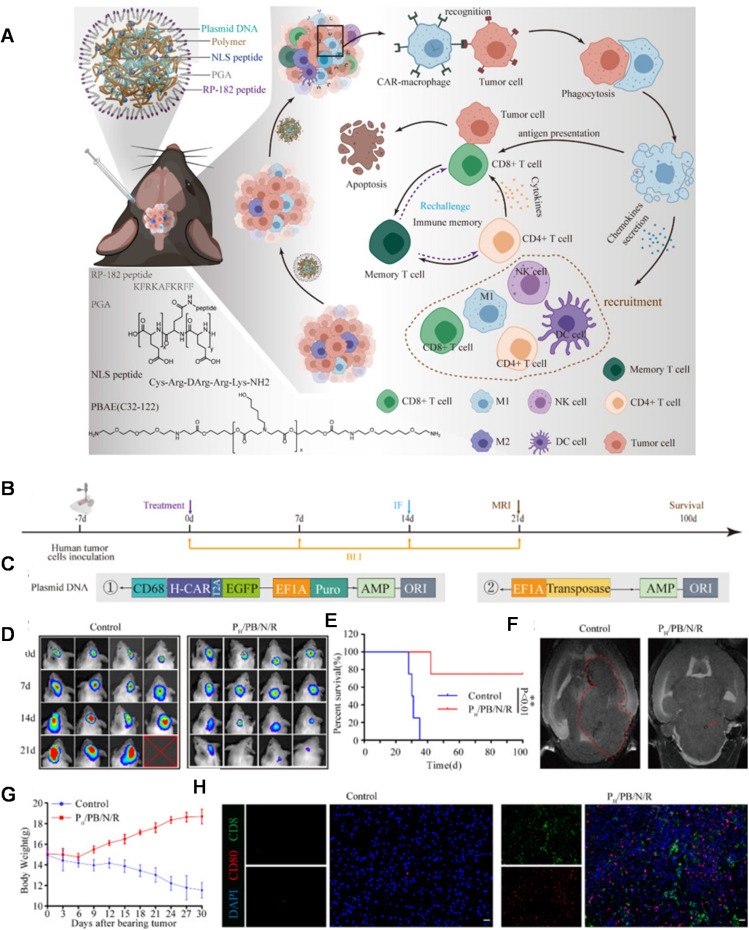
(A) Design and therapeutic mechanisms of macrophage-programming nanoparticles. Additionally, the chemical structures of the PBAE polymer and polyglutamic acid are shown, as well as the amino acid sequence of the nuclear localization (NLS) peptide. (B) Antitumor efficacy of the formulation (named as P_h_/PB/N/R); a nanoparticle formulation comprised of P (polyglutamic acid, PGA) + PB (PBAE polymer) + N (nuclear localization sequence (NLS) conjugated polymer) + R (RP-182 peptide coating) encapsulating a CAR plasmid. In the patient-derived xenograft mouse model (PDX) and schematic illustration of the experimental design. (C) Schematic diagram of the human ErbB2-specific CAR structure. (D) *In Vivo* Imaging System (IVIS) spectrum images of the mice after the formulation P_h_/PB/N/R treatment on different days. (E) Survival curve of the PDX model mice treated with the P_h_/PB/N/R. (F) *In vivo* T2-weighted MR images of tumor-bearing mice after each treatment. Both red circle and arrow in figure point to the tumor site. The photos were processed with ImageJ software. (G) Weight changes in the PDX model mice treated with the formulation. Data are presented as the mean ± SEM (*n* = 3). (H) Representative immunofluorescence images of tumors showing CD8^+^ T cell and CD80^+^ macrophage infiltration in the control and treated groups. Scale bars, 50 µm. Reprinted with permission from. Gao, L. *et al.* (2023)^94^ ‘Convection-enhanced delivery of nanoencapsulated gene locoregionally yielding ErbB2/Her2-specific CAR-macrophages for brainstem glioma immunotherapy’, *Journal of Nanobiotechnology*.

CAR modification of T-cells has been shown as an effective immunotherapy for malignant diseases, however, alternative CAR cells such as CAR macrophages (CAR-M) have garnered recent attention for their enhanced ability to reach and survive in solid tumour microenvironments compared to CAR-T cells. This is due to the higher prevalence of macrophages within these environments and unique effector functions. CAR-M's therefore can reverse the functions of immunosuppressive macrophages within a tumour and also enhance their phagocytic behaviour towards malignant cells. Jiang and co-workers^94^ evaluated the ability for PBAE–pDNA complexes to reprogram macrophages *in situ*, into CAR-M's as a potential treatment for brainstem gliomas, which are primarily paediatric malignancies with extremely poor prognosis (Fig. 4). The authors developed a PBAE based on a 1,4-butanediol diacrylate and 5-aminopentanol backbone, with a nuclear localisation signal peptide conjugated on the pendant chains (named as P_h_/PB/N/R; a nanoparticle formulation comprised of P (polyglutamic acid, PGA) + PB (PBAE polymer) + N (nuclear localization sequence (NLS) conjugated polymer) + R (RP-182 peptide coating)). Similar to Stephan and co-workers,^100^ the authors of this study^94^ also introduced a poly(glutamic acid) coating to express a synthetic analogue of a host defence peptide (RP-182) to induce macrophage targeting and phenotype switching. These components were then co-formulated with pDNA encoding for an ErbB2 (HER2) specific CAR. The authors observed that transfection efficiency of their pDNA PBAE complexes was significantly enhanced by the NLS and RP-182 peptide, with high selectivity for macrophages observed when a CD68 promoter was used in the plasmid construct (Fig. 4). They identified that CAR-M inducing complexes were able to induce a proinflammatory M1 macrophage phenotype, which had antitumour properties on GL261-H glioma cells. These positive results were then replicated *in vivo*, with targeted CAR complexes administered intratumorally, causing full tumour regression in 67% of mice and no significant side effects. Positive outcomes were also seen in a murine orthotopic model for brain stem gliomas, produced from patient-derived brainstem glioma cells using with ErbB2 CAR PBAE complexes administered using convection enhanced delivery. Here, 75% of mice in the treatment group survived at 100 days, compared to the control group which did not survive beyond 40 days.

Another use for PBAEs in cancer immunotherapy applications was reported by Langer and co-workers,^136^ who developed PBAE blends with poly(lactic-*co*-glycolic acid) (PLGA) to enhance the plasmid DNA payload capacity compared to viral vectors. The authors developed a range of 1–10 µm microparticles containing different PBAE content, which they observed that high PBAE contents limited pDNA release whilst 15–25% PBAE was optimum. The microparticles were found to be phagocytosed well by primary human dendritic cells, and the inclusion of the PBAE within the microparticle matrix was found to be essential for gene expression in macrophages, with 25% PBAE microparticles exhibiting 3-fold higher expression than PLGA only particles. Interestingly it was found that the PBAE microparticles also stimulated DCs into a more mature phenotype with surface expression of costimulatory molecules such as F4/80 suggesting that the microparticles may also have an adjuvant effect. To assess the immunogenicity of PBAE-containing microparticle formulations, B6 mice were vaccinated with various formulations containing an SIY peptide antigen encoding DNA and monitored for tumour growth. EL-4 tumours which overexpress SIY showed reduced growth rates and even complete regression in some mice injected with microparticles containing 15% or 25% PBAE, indicating the efficacy of these formulations in inducing anti-tumour immunity.

The above examples have shown the versatility of PBAE chemistry to enable immune cell targeting, and immunotherapy applications. Another key application for DNA in an immunology context is as nucleic acid vaccines, which can encode for a pathogen related antigen and thus initiate an immune response to stimulate protection against the antigen. Although mRNA vaccines have now perhaps overshadowed their DNA counterparts following the COVID-19 pandemic, DNA vaccines can offer significantly greater stability which may be useful outside of cold chain conditions.

The stability of a PBAE based vaccine formulation was shown by Xu and co-workers who developed a lyophilisable nanoparticle formulation containing a COVID-19 DNA vaccine.^137^ The authors previously showed a PEG-PLGA hybrid PBAE system which offered sustained release of reporter gene plasmid over 9 days.^138^ However, in this study they took inspiration from well-established cationic lipids such as C12-200 and DLin-MC3-DMA used in nucleic acid LNP delivery system and developed lipidated PBAEs for their vaccine platform.^137^ For this, they produced highly hydroxylated PBAEs from a backbone of 1,4-butanediol diacrylate and 4-amino butanol which were lipidated using alkyl carboxylic acids (*e.g.* decanoic acid, oleic acid) through enzyme assisted esterification. These highly hydrophobic PBAEs were then co-formulated with PEG-PLGA to as an amphiphilic non-ionic surfactant. C12 containing PBAEs were found to yield the highest transfection efficiency. Remarkably, most formulations were found to yield peak expression after 3 days post treatment, which was attributed to controlled release of the nucleic acid cargo, however no positive control was used to verify these findings. The top performing C12 PBAE also exhibited excellent stability against cryopreservation and lyophilisation when co-formulated with sucrose, with PBAE particles exhibiting comparable transfection efficiency to fresh particles after storage in these conditions for 12 months. Finally, the C12 PBAE was used as a delivery system for a COVID-19 pDNA vaccine, which encoded for the full-length spike protein from the wild-type SARS-CoV-2 virion. Their results showed that the lipidated PBAEs exhibited high, and dose dependent, neutralising antibody titres following storage for 12 months *via* lyophilisation, when vaccinated in a prime boost manner.

We also recently reported the development of a PEGylated PBAE system, based on 1,4-butanediol diacrylate and 5-amino pentanol, which efficiently delivered a plasmid DNA vaccine (Table 1, entry 5).^96^ We investigated the role that PEG density played on the transfection efficiency of pDNA, hence produced a series of formulations containing non-PEGylated, fully PEGylated and semi-PEGylated PBAE polyplexes. Our findings indicated that the PBAE complexes were able to condense pDNA and to transfect dendritic cells efficiently with no tolerability issues. Notably we observed that the semi-PEGylated complexes, produced through mixing PEGylated and non-PEGylated PBAEs, outperformed the other formulations and the lipofectamine positive control. Interestingly the mixed formulation also yielded the highest expression of a bivalent, spike and nucleocapsid encoding, COVID-19 pDNA vaccine.

### Messenger RNA

Endogenous mRNA transfers genetic information between the permanent storage in the genome, and proteins which control cellular and intercellular functions. The transient and therefore non-permanent nature of mRNA has enabled the emergence of new therapeutics such as protein replacement therapies and vaccines, where exogenous mRNA constructs can be administered to produce, in principle, any desired protein. This technology therefore poses many advantages both in terms of manufacture and applicability.^139,140^ As the formulation process for mRNA is, to some extent, sequence agnostic, manufacture of any construct will be identical hence enabling a platform technology. This is an advantage over direct protein therapies, which require unique manufacturing routes for each new protein drug. Accordingly, the development of mRNA systems should take less time from discovery to accessibility in the clinic. mRNA technology has most recently been used to world-changing effect as vaccines for protection against severe COVID-19 disease, by administering mRNA constructs which encode for the SARS-CoV-2 spike protein.^141–143^ The successful large-scale manufacture and global clinical authorisation of Moderna's SpikeVax™ and Pfizer-BioNTech's Comirnaty™ vaccines has initiated a major surge in new mRNA technologies,^128,143^ with over 900 new mRNA therapies under clinical development ranging from vaccines to protein replacement therapies.^144–146^ However, as with other nucleic acid medicines, the susceptibility of mRNA to endogenous nucleases and poor transport into the cytosol for efficient translation into their encoded proteins relies on the use of a delivery vector, typically LNPs. Given the successes of this technology, PBAE's have been explored for delivery of messenger RNA, typically including fundamental studies to identify structure-function relationships to enhance mRNA delivery with PBAEs and development of non-injectable mRNA medicines.^137,147,148^ This section summarises the recent literature on PBAE delivery systems for mRNA therapeutics vaccines for immunology applications.

**Fig. 5:**
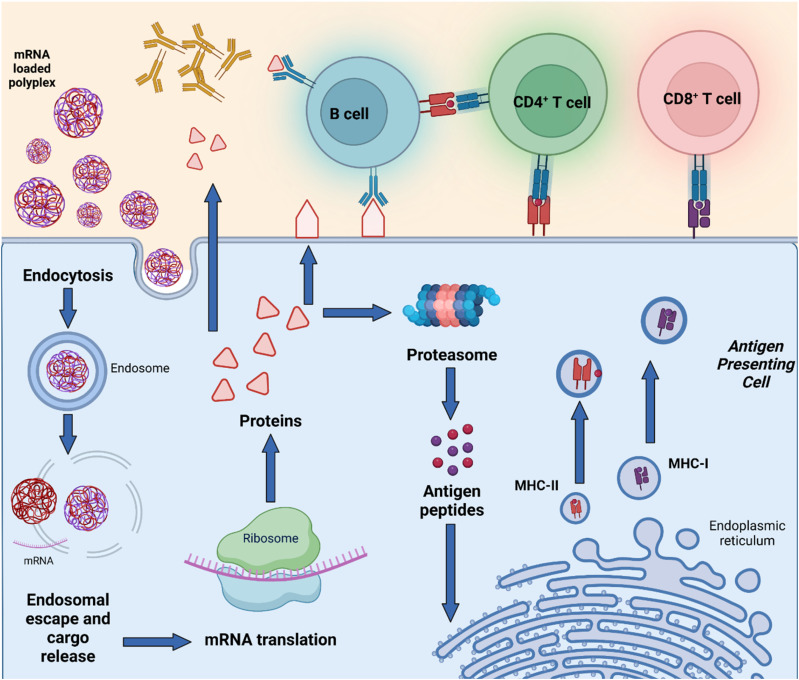
mRNA polyplexes initiate immunotherapy upon endocytosis by APC, followed by endosomal escape and mRNA translation. Resulting proteins are either secreted, expressed on the cell surface, or degraded into peptides. These peptides are then processed for cross-presentation on MHC class I molecules and presented on MHC class II molecules. This presentation activates CD8^+^ and CD4^+^ T cells, respectively. Activated CD4^+^ T cells provide help to B cells, enhancing antibody production and memory formation, while CD8^+^ T cells directly kill infected cells, culminating in a robust and comprehensive immune response.

APCs are a critical part of the adaptive immune system, able to display antigens on their surface and cross-present to other immune cells to eliminate pathogens or produce an antibody response (Fig. 5).^149,150^ Delivery of mRNA antigens to APCs could therefore significantly enhance mRNA vaccine and immunotherapy response.^151^ Borrós and co-workers report the development of oligopeptide end-modified PBAEs (OM-PBAEs) as APC targeting delivery systems for mRNA.^147^ Utilising their established PBAE of 5-aminopentanol copolymerised with 1,4-butanediol diacrylate, they transformed the acrylate end-groups with a further aza-Michael addition of a series of oligopeptides containing cysteine, histidine and lysine. When formulated with mRNA these OM-PBAEs they were able to produce small polyplex nanoparticles, approximately 140 nm in diameter, which significantly enhanced transfection efficiency of eGFP encoding mRNA in HeLa, ARPE-19, RAW264.7 and JAWSII cell lines. After intravenous administration, the authors reported excellent accumulation in the spleen with OM-PBAEs, and high uptake (through Cy5 labelled PBAEs) in splenic macrophages, dendritic cells, and neutrophils. Despite this, relatively modest transfection efficiency was observed in these isolated cells. One potential limitation of their study is that polyplex formulations often contain a high proportion of non-complexed polymer, due the requirement of high N/P ratios to form low particle sizes hence the uptake studies may not be accurately correlating the nanoparticle uptake and transfection.

To enhance further APC targeting, the authors reported a follow up study in which they developed OM-PBAEs containing mannose pendant chains with the objective to improve uptake through mannose receptor mediated endocytosis.^152^ Through careful formulation with mRNA, these polymers yielded nanoparticles adorned with surface-exposed α-mannose molecules, measuring approximately 180 nm in size, and possessing a positive surface charge. Importantly, these nanoparticles maintained their characteristics even after freeze-drying and subsequent rehydration. The uptake of mannose expressing mRNA carrying PBAEs in JAWSII cells was found to be significantly reduced when free mannose was co-incubated, indicating competition and mannose receptor dependent uptake. Despite the improvement in uptake with increasing mannose content, it was found that this did not markedly increase transfection efficiencies. In fact, significantly reduced mRNA expression was observed, highlighting that structural adaptations to increase nanoparticle uptake alone may influence other aspects of delivery. Nonetheless the synthetic approach introduced here could be adapted to produce libraries of glycosylated PBAEs, although further work is needed to ensure high transfection efficiencies are observed.

As described above, therapies based on modulation of immune cells have now reached clinical approval utilising DNA based reprogramming of T-cells, either through CAR-T or T-cell receptor modifications. However, there is now a shift to manipulate these cells transiently using mRNA gene expression. Accordingly, Stephan and co-workers^153^ utilised their poly(glutamic acid) (PGA) antibody coating approach described in the DNA section, to decorate mRNA loaded PBAE complexes with a variety of antibodies (anti-CD3, anti-CD4, anti-CD8, anti-CD28 and control antibody C1.18.4). The polyanionic PGA coating also serves to reduce non-specific electrostatic interactions between positively charged polyplexes and the electrostatic membrane of non-target cells. To exemplify this PBAE platform, the authors found that the antibody-coated PBAE polyplexes exhibited a tenfold increase in *ex vivo* T-cell transfection, compared to non-targeted PBAE NPs. Expanding on this success, the versatile platform was utilized for the delivery of two different mRNAs. In one instance, these NPs delivered megaTAL nuclease mRNA to disable endogenous T-cell receptors potentially implicated in graft-versus-host disease. In a separate application, NPs loaded with mRNA encoding the Foxo13A transcription factor were employed to guide CD62L^+^ T-cells away from terminal differentiation and senescence, promoting a central memory phenotype. Collectively, these outcomes demonstrate the adaptability of the mRNA platform, which can use the same PBAE for different therapeutic applications varying only the sequence of the mRNA and with no change in the formulation process.

**Fig. 6:**
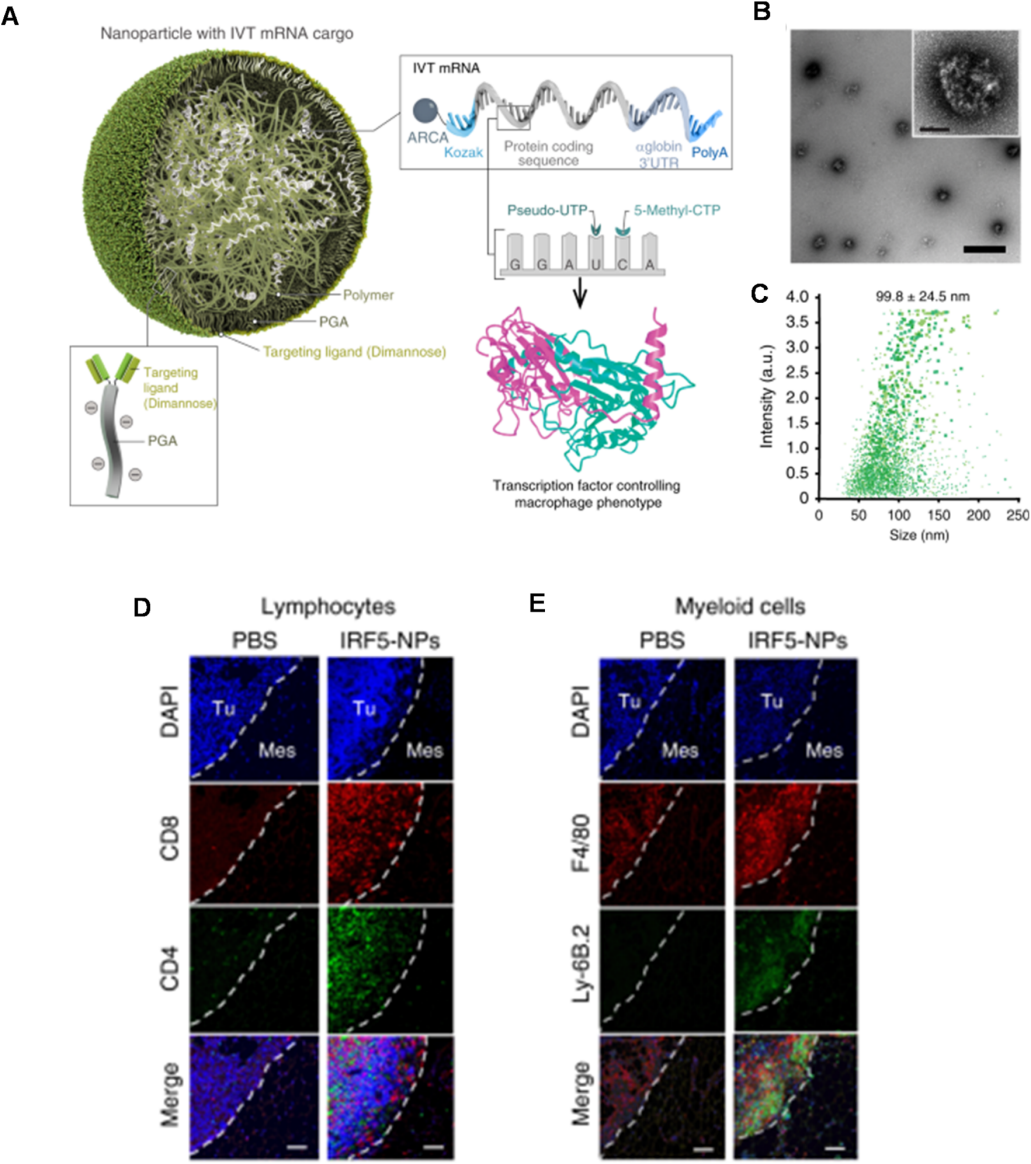
(A) Mannose receptor-targeted mRNA nanoparticles efficiently transfect M2 macrophages. Design of macrophage-targeted polymeric NPs formulated with mRNAs encoding key regulators of macrophage polarization. The particles consist of a PBAE–mRNA polyplex core coated with a layer of PGA-Di-mannose, which targets the particles to mannose receptors (CD206) expressed by M2-like macrophages. Also depicted is the synthetic mRNA encapsulated in the NP, which is engineered to encode the reprogramming transcription factors. (B) Transmission electron microscopy of a population of NPs (scale bar 200 nm) and a single NP (inset, scale bar 50 nm). (C) Size distributions measured using a NanoSight NS300 instrument. (D and E) T cells contribute to anti-tumor effects achieved with macrophage-programming nanoparticles. Nanoparticle-mediated macrophage programming increases T cell recruitment into tumor lesions. Shown are representative confocal images of peritoneal metastases of ID8 ovarian cancer cells in the mesentery. Tissues were collected after 6 biweekly i.p. injections of PBS or IRF5/IKKβ NPs (50 µg mRNA/dose) and were stained for the indicated lymphocyte- and myeloid-markers. Tu = Tumor, Mes = Mesentery. Scale bar: 100 µm. Reprinted with permission from. Zhang, F. *et al.* (2019)^4^ ‘Genetic programming of macrophages to perform anti-tumor functions using targeted mRNA nanocarriers’, *Nature communications*.

The same group also conducted a follow up study utilising the PGA coated PBAE complex approach containing, but instead harbouring a di-mannose ligand on the surface to target the mannose receptor, overexpressed on macrophages (Table 1, entry 7).^4^ They then used this system to deliver two mRNA constructs; one encoding IRF5, a member of the interferon regulatory factor family that stimulates polarisation of macrophages towards the M1 phenotype and an mRNA encoding for IKKb a kinase which phosphorylates and activates IRF5 (Fig. 6). As discussed in the DNA section above, M1 phenotype macrophages have the potential to phagocytose pathogens and have anti-tumour properties, unlike M2 macrophages which decrease inflammatory response and therefore are pro-tumoural. The authors hypothesised that repolarising M2 macrophages using their mRNA delivery system could act as a tumour regression therapy. It was found that, following treatment of M2 macrophages with the repolarising mRNA complexes, these macrophage cells displayed heavily downregulated signature M2 macrophages, whilst M1 differentiation genes were upregulated. In a model which mimics unresectable ovarian tumours, 40% of mice which received the repolarising PBAE complexes locally to tumours exhibited complete elimination of disease (142 days median survival compared to 60 days in control). Interestingly the authors also found that the IRF5/IKKb PBAE complexes increased T cell infiltration 10.6-fold for CD8^+^ and 3.5-fold for CD4^+^ T cells which likely contributed to some of the anti-tumour potency seen, which was further confirmed through CD8^+^ T cell suppression with a monoclonal antibody. For further clinical applicability of this system, the authors also demonstrated that i.v administration led to accumulation in macrophage rich organs (spleen, liver, lungs) and displayed efficient tumour regression in a murine model for pulmonary melanoma metastatic cancer. These successful outcomes were also replicated in a murine glioma model, a cancer where M2 like macrophages represent a considerable proportion of neoplastic cells. Nine doses of i.v administered IRF/IKKb PBAE complexes only displayed modest tumour progression, however when adjuvanted with radiotherapy as the standard of care for gliomas, the PBAE complexes group more than doubled the survival of treated mice compared to the control group (52 days compared to 25 days). This pioneering study shows the potential for PBAE engineering to enhance delivery of complex immunotherapies which can be applied to many hard-to-treat malignant diseases.

A critical aspect for the design of a delivery system, including for immune cells, is the tolerability of the vector. The polycationic nature of PBAEs however can disrupt negatively charged cell membranes inducing an undesired response. In the case of vaccines and immunotherapies, this inflammatory response may be beneficial, however for therapeutic strategies minimising toxicity is usually considered essential. Irvine and co-workers^102^ developed PBAE nanoparticles coated in a phospholipid bilayer to minimise interaction with the cell membrane, and mRNA was then adsorbed to the corona of these hybrid nanoparticles. For their PBAE polymers they built on previous work by Lynn and co-workers^33^ and used a 1,4-butanediol diacrylate copolymerised with a bis piperidine monomer, designed to promote endolysosomal escape. Particles were produced either through direct formulation in ethanol, followed by dialysis or a double emulsion technique utilising dichloromethane, with both approaches yielding similar nanoparticle sizes. In further *in vitro* studies the authors demonstrated the ability for these hybrid formulations to induce endosomal escape in DC2.4 dendritic cells. Using Annexin V staining, all lipid-enveloped PBAE nanoparticles showed lower toxicities than non-lipid coated formulations, at 50–100 µg mL^−1^ nanoparticle concentrations after 1 h and 12 h of treatment. When formulated with eGFP encoding mRNA, lipid enveloped formulations showed 30% of dendritic cells expressing eGFP, comparable to many commercial non-viral vectors in this cell line. Finally, the lipid coated PBAE formulations displayed substantially improved delivery and gene expression, compared to non-formulated mRNA when administered intranasally. However, no comparison between lipid enveloped and non-lipid enveloped mRNA was made. These results highlight the versatility of PBAE systems, and their ability to incorporate other ‘off the shelf’ excipients to boost performance.

The hybrid PBAE–lipid nanoparticle approach has also been investigated for wider delivery applications, including to the lungs, where many immune cells reside, such as alveolar macrophages which form part of our innate immune system. For instance, Anderson and co-workers^106–108^ have reported several studies in this area. Here, we highlight one of their key studies focusing on optimisation of PBAE chemistry for delivery to pulmonary immune cells and lung epithelium. The authors produced a library of PBAEs based on a bisphenol A glycerate diacrylate and 4-(2-amino methyl) morpholine polymer backbone containing different lengths of alkylamine comonomer and various endcaps (Table 1, entry 15). Formulations were then co-formulated with mRNA, a range of PEG lipids, phospholipid and cholesterol to produce PBAE lipid hybrids. Given the large formulation space available with these components, a multifactorial experimental design screen was conducted showing the highest transfection efficiency in HeLa cells arising from a C10 alkyl amine co-monomer, and diamino propane end group containing a 7 wt% content of C14-PEG2000 PEGylated lipid. A further partial factorial screen was conducted to identify formulations with higher lung specificity, which showed that hybrid particles containing C18-PEG2000 exhibited high lung specificity. Subsequent investigation demonstrated that when administered intravenously, the lung targeted formulations yielded mRNA expression in 70% of pulmonary epithelial cells. Analysis of the immune cell reaction showed the highest proportion of transfection within dendritic cells, B cell and alveolar monocytes.

As discussed above, cancer immunotherapy applications using PBAEs to deliver genetic material to APCs and T-cells have been widely studied.^102,154,155^ However, many of these reports have utilised ligands to enhance association with immune cells. Green and co-workers^156^ reported the direct optimisation of lipid containing bioreducible PBAEs to enhance delivery of a prostate cancer antigen to dendritic cells, using the intrinsic properties of the PBAE itself. First a small combinatorial library of PBAEs containing C12–C18 amino co-monomers copolymerised with 4-amino butanol, a disulfide containing diacrylate (for cytosol degradation through glutathione), and a variety of end capping amines were produced. All formulations efficiently entrapped mRNA producing PBAE polyplex nanoparticles below 150 nm in diameter and positive (*ca.* +20 mV) zeta potentials. Notably, the most lipophilic PBAE produced the highest transfection efficiency, with over 75% of DC2.4 cells expressing the eGFP reporter gene. A key innovation in this study was also the inclusion of other nucleic adjuvants co-formulated within the PBAE complexes to boost the immune response and ideally trigger greater presentation of the antigen to cytotoxic T lymphocytes. The co-formulation approach also ensures that the mRNA cargo and immunostimulatory adjuvant reach the same cells. In this study the authors co-formulated agonists for Toll-like receptors (TLR), including polyI:C, a dsRNA TLR3 agonist and CpG oligodinucleotides (ODNs), a dsDNA TLR9 agonist. The mechanism behind these adjuvants is discussed below in the oligonucleotides section. Notably inclusion of CpG ODNs did not influence transfection efficiency, while formulations incorporating polyI:C displayed a 50% reduction in mRNA expressing cells. As in DC2.4 cells, the most lipophilic formulations also exhibited the highest expression of luciferase encoding mRNA in more relevant murine bone marrow derived dendritic cells. Similarly, in further animal studies, the authors showed that upon systemic administration, lipophillic PBAEs accumulated in the spleen, with high transfection efficiency of splenic DCs and high expression of DC activation markers, CD40 and CD86 when co-formulated with polyI:C and CpG ODNs. Finally, the hit formulation also displayed significantly reduced tumour burden when used as a delivery system for mRNA encoding for ovalbumin and F10 antigens to initiate an immune response.

Another example of adjuvantation of mRNA encoded antigens was reported by Gu and co-workers^60^ who utilised branched PBAEs based on a bisphenol A ethoxylated diacrylate and 4-aminobutanol backbone branched using trimethylolpropane triacrylate, co-formulated with iron oxide nanoparticles. Interestingly addition of the iron oxide nanoparticles did not influence transfection efficiency in BMDCs but an 8–10% reduction was noted in DC2.4's. Further studies using ovalbumin antigen encoding mRNA showed formulations with and without iron oxide nanoparticles caused upregulation of CD86 and MHCII maturation markers in BMDCs. However, the iron oxide containing PBAE complexes exhibited significantly higher pro-inflammatory cytokine expression (IL-6 and TNFa), highlighting the importance of the adjuvant excipients. When evaluated in an *in vivo* tumour model expressing OVA antigen, mice treated with the PBAE OVA vaccine showed almost complete tumour growth inhibition (200 mm^3^ tumour volume for vaccine compared to 1200 mm^3^ PBS control), compared to the non-adjuvanted formulation. Finally, the authors also demonstrated that the iron oxide formulation showed improved efficacy in larger tumours when co-injected with anti-PD1 immune checkpoint inhibitors. This study highlights the versatility of PBAEs to incorporate a range of excipient cargos to boost immune response for desired applications.

Whilst the lung, spleen and lungs are considered immune rich organs, the gut is also highly active with immune rich compartments such as Peyer's patches. The gut-immune system is also responsible for several inflammatory disorders and a convenient yet effective route for vaccination against infectious diseases (*e.g.* cholera, polio, rotavirus and typhoid) due to the immune rich mucosa, offering systemic and mucosal immunity. However, the low pH and destructive biological environment have been a substantial challenge for oral delivery of more complex biologics including mRNA vaccines. Traverso and co-workers^105^ reported on the development of PBAE formulations to deliver mRNA antigens *via* the oral route. First, they conducted a screen on a branched PBAE library, and identified a hit polymer (Table 1, entry 14) exhibiting high transfection in Caco-2 cells, which were further evaluated in BMDCs. However, a distinct limitation with this study is the minimal discussion around the structures of these polymers in this paper and previous studies,^157^ hence it is difficult to draw conclusions in terms of structure function relationships. Their preferred candidate PBAE 844 (as named in the original study), showed a 30% increase in CD22^+^ B-cells and 100% increase in CD69^+^ B cells indicating their activation compared to a PBS control when administered through surgical administration to the small intestine. Furthermore, a modest increase in activated T-cells was also observed. However, when administered through oral gavage, both B-cell and T-cell activation was negligible and no antigen expression was observed, further highlighting the challenges associated with oral administration, but also the opportunity for oral mRNA vaccines in future.

Most non-viral delivery systems for mRNA typically use liquid formulations to be administered by simple injections. However, many applications including tissue engineering or administration during surgical intervention could require implantation of delivery devices. Bryers and co-workers^158^ reported an elegant study reporting the development of mRNA complex loaded poly(hydroxy ethyl methacrylate) (pHEMA) porous scaffolds. Their scaffolds were engineered by utilising poly(methyl methacrylate) (PMMA) microspheres to template pores into the pHEMA matrix, which were removed later through dissolution in acetone. While the authors did initially evaluate a range of non-viral vectors for their transfection efficiencies in 2D cultures, the PBAE was not taken forward due to its lower activity in DC2.4's and BMDCs compared to commercially available lipid-based vector, StemFect™. Whilst StemFect™ loaded hydrogels exhibited sustained expression of eGFP encoding mRNA, given the scope of this review, understanding the performance of PBAE vectors using this platform would be of interest to the community.

### Self-amplifying mRNA

mRNA vaccines and therapeutics have revolutionised our approach to drug and vaccine development, however the short-lived expression of exogenously delivered mRNA represents a significant challenge for therapeutics which require repeat dosing, whilst the high dose requirements also create a high-cost burden for these new medicines. Innovations in mRNA construct design, such as self-amplifying mRNA (saRNA) have been shown to alleviate some of these early challenges.^159–161^ Self-amplifying mRNAs operate by incorporating non-structural proteins which comprise the replication complex derived from alphaviruses into the coding sequence of the mRNA constructs.^162,163^ These are expressed alongside the desired protein/antigen, which recognise the residual saRNA and amplify the sequence, producing further replicases.^162^ This cycle repeats itself exponentially, enhancing the expression lifetime of saRNA to over 60 days compared to the 2–7 days lifetime of traditional mRNA.^164,165^ These advantages also enable substantially lower doses, with studies indicating that saRNA vaccines can yield similar levels of protective immunity to mRNA vaccines at greater than 100-fold lower doses also reducing the cost burden of this technology.^159,164^ Despite initially poor results for saRNA in several clinical trials, Japan became the first country to approve an saRNA vaccine (ARCT-154) for protection against severe COVID-19 disease, developed by Arcturus Therapeutics. However, saRNA constructs can be 5–10-fold greater in length compared to conventional mRNA, reaching up to 12 000 nt, which increases their vulnerability to endonuclease degradation, and accordingly can require modification of the formulation parameters.^164,166^ Furthermore, the limited ability to date to incorporate modified bases also potentiates their formulation requirements. Whilst there are now many examples in the literature using lipid nanoparticles and other polymeric non-viral vectors for saRNA, there remain relatively few examples of PBAEs used for saRNA.^109,167^

**Fig. 7:**
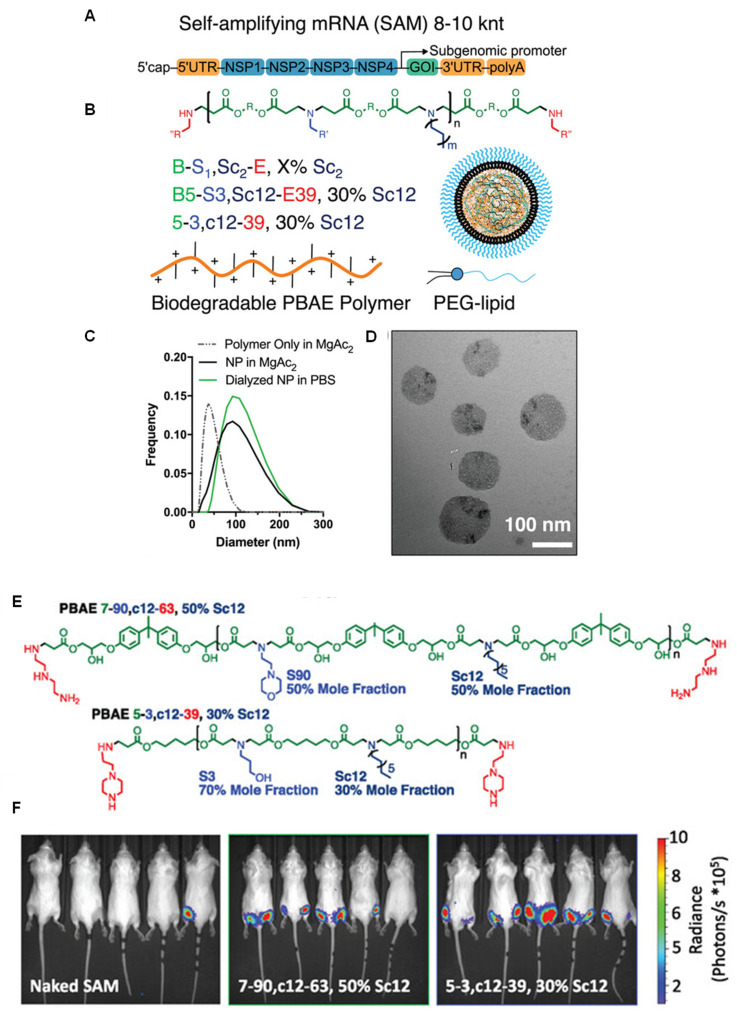
(A) SAM structure including a 5′ cap, 5′ untranslated region (UTR), non-structural protein genes 1–4 from alphavirus, GOI, 3′ UTR, and PolyA tail. (B) Generalized structure of PBAE polymer, naming scheme for 4-component polymer and cartoon of assembled nanoparticle with PEG–lipid (C) DLS measurement of polymeric nanoparticles with and without SAM. (D) TEM microscopy of SAM nanoparticles. (E) Structures of lead polymers for intramuscular administration. (F) Representative IVIS images with the top two nanoparticle formulations compared to naked SAM. Reprinted with permission from. Wilson, D. R. *et al.* (2023)^109^ ‘Biodegradable Polyester Nanoparticle Vaccines Deliver Self-Amplifying mRNA in Mice at Low Doses’, *Adv Ther* (Weinh).

Green and co-workers^109^ used self-amplifying mRNA to significant effect by highlighting their immense potential as low dose vaccines, delivered using PBAEs. First, a semi-high throughput approach was conducted to produce 196 unique polymers with varying backbone monomers, pendant amines, and end caps, which were further tests for transfection efficiency of self-amplifying mRNA *in vitro* (Fig. 7). As with previous studies, they identified that inclusion of higher mole fractions of lipophilic monomers and end caps containing ionisable amines significantly enhanced expression efficiency even as low as 1 ng per well, 100–200-fold lower than the conventional dose of mRNA. Interestingly, the authors showed that co-formulation with DMG PEG2000, a common PEGylated lipid, was essential for mRNA expression when administered intramuscularly, attributed to improved extracellular transport. Their optimised PBAEs for were also potent delivery systems for rabies virus glycoprotein saRNA vaccine constructs. Mice vaccinated with 100 ng saRNA exhibited high neutralising antibody titers after challenge with live rabies virus, significantly above 0.5 IU mL^−1^ which is considered as the level required for protective immunity.

We also recently reported the use of PBAE complexes for delivery of saRNA.^167^ As discussed above one of the critical challenges for polycation use as non-viral vectors is to minimise disruption of the cell membrane through charge mediated interaction. We therefore investigated this by incorporating a polyanion, polyglutamic acid, into saRNA PBAE complexes in two different ways, coating preformed complexes or mixing all three components in one step.^167^ PBAE-based polymers were prepared from 1,6-hexanedioldiacrylate and 4-aminobutanol. Our results demonstrate that incorporating γ-PGA into polyplexes enhanced transfection efficacy in HEK293T and A431 cells, while maintaining polyplex size. The addition of PGA also, as expected, resulted in a significant decrease in zeta potential, reducing the toxicity of the ternary complexes in moDC, NIH3T3, and A431 cells. Furthermore, the presence of γ-PGA contributed to colloidal stability, reducing the aggregation of ternary complexes, as evidenced by insignificant changes in the polydispersity index (PDI) after freeze–thaw cycles.

### Therapeutic oligonucleotides

Oligonucleotides are short nucleic acid sequences which have been primarily investigated as regulators of gene expression. These operate by selectively binding to complementary mRNA sequences, a process known as RNA interference (RNAi), to limit the expression of disease related proteins and classically ‘undruggable’ targets. Oligonucleotides exist in different classes, such as small interfering RNAs (siRNA), microRNAs (miRNA) and antisense oligonucleotides (ASO) each displaying unique mechanisms for RNA interference.^168,169^ Furthermore, other oligonucleotides such as short guide RNAs are critical for the function of CRISPR-Cas gene editing technologies. In an immunology context, endogenous microRNAs modulate inflammation and immunity in response to pathogens, due to their role in cell–cell communication,^170^ hence have garnered interest as a route to modulate the immune system. Furthermore, the solid phase synthesis methodologies for these oligomeric nucleic acids means that base, backbone, and sugar modifications can be made with ease to tune stability and affinity.^171^

Similar to other nucleic acid cargo's, oligonucleotides also require a delivery vector (*e.g.* polycations, lipid nanoparticles *etc.*) or modifications (such as GalNac conjugation) to improve uptake by target cells.^172–175^ Although there are many examples of PBAE based oligonucleotide delivery as potential cancer therapeutics, their use in immunology contexts have been scarce. During our search of the literature, we did not find any examples of PBAE delivery systems for siRNA, miRNA and ASO therapies for immunology applications. Yet other non-viral delivery systems (*e.g.* LNPs, PLGA, chitosan and PEI) have been explored for haematological cancers, as immune checkpoint silencers and for reprogramming the phenotype of macrophages, dendritic cells, and T-cells.^173,175,176^ Given the high tuneability of PBAEs seen for other immune related applications, and in particular, developments of organ targeted and cell specific chemistries, we believe there is significant scope for their use in these applications. In principle, these new frontiers for PBAEs should be relatively achievable as there is now a strong body of work showing PBAE delivery systems which can co-deliver oligonucleotide adjuvants with protein, DNA, or mRNA antigens. However, as our review is structured by cargo class, examples of oligonucleotide adjuvants can be found with those studies or in the section below when administered independently.

### Delivery of nucleic acid adjuvants

Although the first concepts of immunisation against infectious disease can be traced back to 900 AD China, the focus over the last 200 years in vaccine development has shifted to improving vaccine safety without compromising on durability of protection. The most common approach to achieve this has been to incorporate adjuvants, materials which can stimulate responses to infection processes, allowing the use of safer, purified antigens. An emerging class of adjuvants are non-coding nucleic acids, which mimic the natural infection process of DNA and mRNA viruses by stimulating pathogen recognition receptors (PRRs), typically Toll-like receptors (*e.g.* TLR3, 7, 8 and 9) and cytosolic sensors (*e.g.* MDA-5), to initiate expression of pro-inflammatory cytokines to boost the immunocompetency at the injection site. Adjuvants are also critical to the function of immunotherapies, particularly for enhancing the cytotoxic response of CD8^+^ T-cells in immuno-oncology applications. Given the superior performance of PBAEs in delivering coding nucleic acids, there have also been several reports of PBAEs as delivery system for nucleic acid adjuvants. In this section, we include examples where nucleic acid adjuvants have been administered independent of a protein, or nucleic acid antigen, which can be found in their associated sections.

Jewell and colleagues^104^ investigated the potential of PBAEs for delivering toll-like receptor agonists (TLRas), specifically CG rich oligodinucleotides (CpG-ODNs), to enhance immune responses for potential use in immuno-oncology applications (Table 1, entry 13). Given the cationic nature of PBAEs, these were able to self-assemble with CpG-ODNs to form polyplexes. Surprisingly, it was found that polyplexes with lower PBAE : CpG ratios, despite weaker CpG condensation, induced more effective activation of dendritic cells and tumor-specific T cells compared to higher ratios, leading to improved survival with mean values of 14 ± 5 days in a mouse melanoma model. This highlights the crucial role of physicochemical properties, particularly the interplay between charge, uptake, and affinity, in modulating the efficacy of the generated immune response. The research highlights also the importance of Toll-like receptors (TLRs) as targets for enhancing immunotherapy potency and emphasizes the need to balance nanoparticle characteristics for optimal design, offering valuable insights for developing new adjuvant carriers for vaccines and immunotherapies.

Another TLR agonist which has been widely investigated as a potential adjuvant for vaccines is polyI:C, a non-coding dsRNA nucleic acid polymer of inosine and cytosine. polyI:C is also able to activate cytosolic RNA sensors (*e.g.* RIG-I and MDA-5) to push target DCs into an antiviral state and initiate expression of type I interferon. However as with other nucleic acids and adjuvants, transportation of polyI:C to DCs is critical for its efficacy. It should also be mentioned that polyI:C is currently under clinical trials, known as ampligen, for myalgic encephalomyelitis using poly-l-lysine as a delivery system. Appel and co-workers^110^ reported optimisation of PBAEs for delivery of polyI:C (Table 1, entry 17). In this study, the authors systematically characterized and screened a library of pIC/PBAE NPs for increased potency and reduced toxicity and evaluate the *in vivo* efficacy of pIC/PBAE NPs in a model subunit vaccine. They identified PEG-coated pIC/PBAE NP (2B) formulation that enhanced type I IFN production by 13-fold compared to pIC alone *in vivo* which significantly improves the magnitude, duration, and affinity maturation of antigen-specific antibodies following vaccination.

### Delivery of chemotherapeutic molecules

PBAEs have been widely studied as agents to complex nucleic acids for delivery owing to their cationic charge. However, this feature, and also their versatility in end-group and pendant chain chemistries, also favours PBAEs for small molecule delivery. Whilst this has been explored for conventional cancer therapeutics and antimicrobial agents, we identified only one example of small molecule delivery in an immunotherapy context despite the existence of many immunomodulatory chemotherapeutics such as imidazoquinolines. Green and co-workers^99^ showed a crucial advancement in cancer immunotherapy by utilising the capabilities of PBAE-based nanomedicine for the efficient delivery of cyclic dinucleotides (Table 1, entry 8). Tumour microenvironments can produce an immunosuppressive environment, and hence creating a pro-inflammatory environment with small molecule adjuvants may aid immunotherapy applications. Some small molecule cyclic dinucleotides (CDNs) have been shown to be potent Stimulator of Interferon Gene receptor (STING) agonists, and are presently undergoing phase I trials. However, their effectiveness might be constrained to micromolar concentrations because STING resides in the cytosol within the endoplasmic reticulum membrane. Despite the challenge of the small size of CDNs, their negative charge facilitates effective encapsulation by cationic PBAEs. For the CDN PBAE nanoparticle formulation, 1,4-butanediol diacrylate and 4-amino-1-butanol monomers were used to prepare the polymer core followed by end-capping with 1-(3-aminopropyl)-4-methylpiperazine. These nanoparticles, characterized by a modestly positive surface charge and a diameter of approximately 100 nm, exhibited efficient cellular uptake by THP1 human monocyte cell lines and human donor monocyte samples, suggesting their potential for targeted delivery to immune cells within the complex tumour microenvironment. In addition, through this innovative approach, a large increase in potency (around 100-fold) was observed when treating established B16 melanoma tumours *in vivo*, particularly when combined with a PD-1 blocking antibody, compared to free CDNs without nanoparticles. Overcoming the inherent challenges posed by the small size of CDN molecules, this study displays a promising strategy for enhancing the efficacy of cancer immunotherapy through precise and efficient nanoparticle-mediated delivery of immunomodulatory agents. Overall, while PBAE-based nanoparticles hold great potential for small molecule drug delivery, further research and optimisation are needed for immune-based treatments to understand fully their benefits in clinical settings.

### PBAE functionalization and immune-targeted strategies

PBAEs can be modified by a range of chemistries in the backbone, in the pendant chains, or at end-groups, and can also be co-formulated to achieve selective delivery to specific immune cell subsets.

**Fig. 8:**
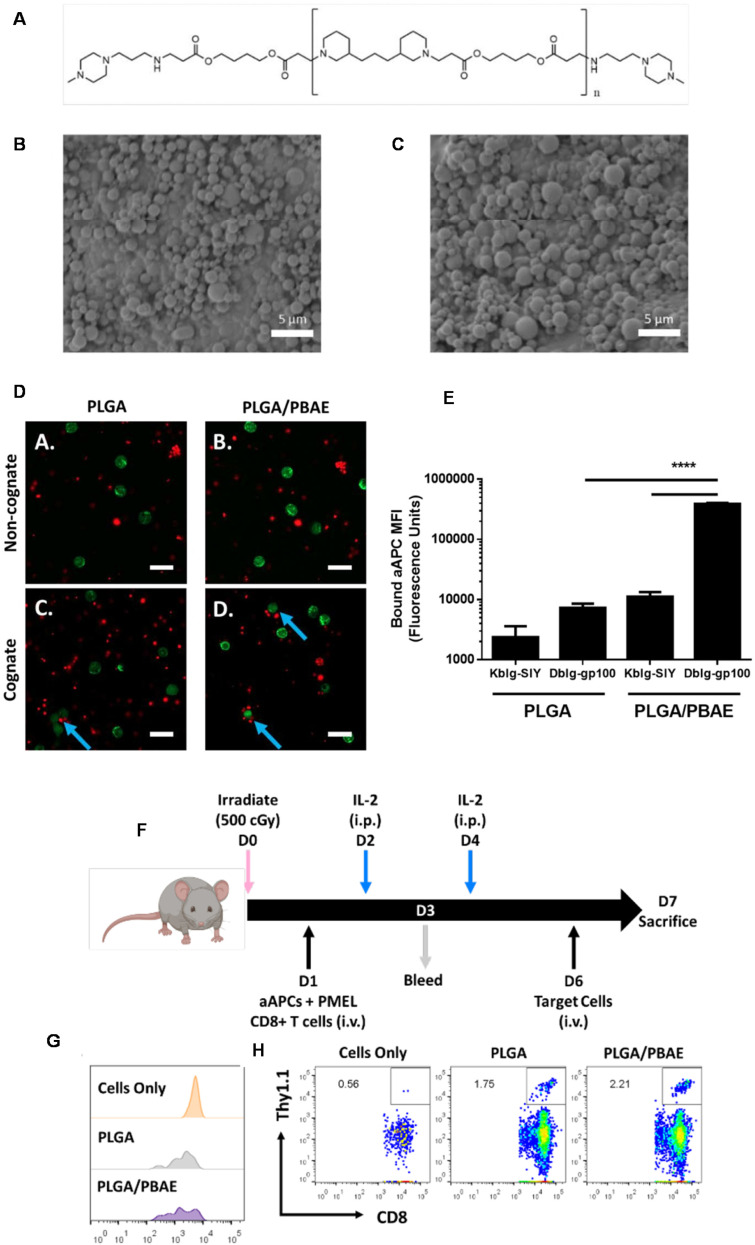
(A) Chemical structure of PBAE synthesized *via* Michael addition of 1,4-butanediol diacrylate and 4,4-trimethylenedipiperidine and endcapped with 1-(3-aminopropyl)-4-methylpiperazine. SEM micrographs of (B) PLGA and (C) PLGA/PBAE microparticles. (D) PLGA/PBAE aAPC (red) show higher levels of binding to cognate CD8^+^ T cells (green) compared to PLGA aAPC. DiD-labelled MHC DbIg-gp100 (cognate) or MHC KbIg-SIY (noncognate) aAPC were incubated with CFSE-labelled PMEL CD8^+^ T cells for 1 hour at 37 °C. Representative confocal images illustrate (D-A) PLGA and (D-B) PLGA/PBAE aAPC exhibit low levels of binding to non-cognate CD8^+^ T cells. (D-C) PLGA aAPC show relatively low levels of binding to cognate CD8^+^ T cells. Blue arrows indicate binding events. (D-D) PLGA/PBAE aAPC bind more frequently and in higher numbers to cognate-CD8^+^ T cells. Scale bars = 20 µm. (E) After incubation of CD8^+^ T cells with particles, cells were washed and analyzed using flow cytometry. DiD fluorescence intensity was normalized to control samples with blank particles. PLGA/PBAE aAPC demonstrate enhanced binding to cognate CD8^+^ T cells, as evidenced by a more than 50-fold increase in DiD fluorescence intensity over CD8^+^ T cells incubated with PLGA aAPC. Error bars represent the SEM of 4 replicates, (* = *p* < 0.05, ** = *p* < 0.01, *** = *p* < 0.001, **** = *p* < 0.0001). (F) Schematic of *in vivo* antigen-specific CD8^+^ T cell expansion and killing assay timeline. Representative (G) CTV dilution in PMEL cells in the blood on day 3 and (H) FACS plots showing the percentage of Thy1.1^+^ CD8^+^ cells in the spleen on day 7. Reprinted with permission from. Rhodes, K. R. *et al.* (2022)^101^ ‘Biodegradable Cationic Polymer Blends for Fabrication of Enhanced Artificial Antigen Presenting Cells to Treat Melanoma’, *ACS Appl Mater Interfaces*.

Green and co-workers^101^ demonstrated a co-formulation approach, combining PBAEs with PLGA microparticles to display MHC proteins and co-stimulatory molecules (CD28), generating artificial antigen-presenting cells (aAPCs) (Fig. 8). For these microparticles a PBAE polymer comprising 4,4′-trimethylenedipiperidine and 1,4-butanediol diacrylate (Table 1, entry 10) was co-formulated with PLGA, which was further functionalised with a range of antigens MHC's (KbIg-SI, DbIg-gp100 and KbIg-TRp2) and anti-CD28 to facilitate T-cell binding. They observed that conjugation of these proteins on PBAE containing microparticles was up to 89 times more effective than PLGA only microparticles. The resulting PLGA/PBAE aAPCs demonstrated improved T cell activation and expansion both *ex vivo* and *in vivo* in B16–F10 melanoma mouse model without the need for adoptive transfer, showing efficacy in reducing tumour growth and extending survival in a melanoma mouse model when combined with checkpoint therapy. The authors discuss that the intrinsic immunostimulatory effects of the PBAE may also have also contributed to its performance in this application, highlighting the potential broad use of PBAEs in immunology.

Recently, Wang and co-workers engineered a PBAE-based direct polymer functionalization strategy by synthesizing lentinan-functionalized PBAE-G nanodiamond (LNT-PBAE-G-ND) polymers.^177^ In this study, they developed PBAE-guanidine-phenylboronic acid (PBAE-G) polymers and termed the system as Lentinan-Functionalised PBAE-G-nanodiamond (LNT-PBAE-G-ND). They delivered mRNA (OVA) and evaluated the impact of this delivery system on macrophage activation and maturation. *In vitro* experiments revealed that LNT-PBAE-G-ND@OVA successfully be transfected into various cell lines and enhanced the immune response (analysing IL-1β, IL-6, IL-12, and TNF-α) relative to the nontreated group (*P* < 0.05), reaching levels comparable to those in the LPS positive control group (*P* > 0.05). Furthermore, the *in vivo* investigations demonstrated that intramuscular administration of LNT-PBAE-G-ND@OVA efficiently delivered the vaccine, eliciting robust humoral and cellular immune responses (IgG1 and IgG2 levels were *P* < 0.01) without evident toxicity which was confirmed *via* histopathology and biochemistry analysis. As a result of these findings, they suggested that LNT-PBAE-G-ND@OVA can induce macrophage activation wherein the cGAS-STING signaling pathway plays a crucial role.

To exemplify the versatility of PBAE functionalization strategies, Artzi and colleagues^178^ have highlighted the immunomodulatory potential of PBAEs by using them to systemically deliver covalently conjugated STING agonists, specifically cyclic dinucleotides (CDNs), *via* PBAE nanoparticles. These CDN-conjugated nanoparticles exhibited improved pharmacokinetics, with plasma detection up to 1 hour post-injection (*vs.* <15 minutes for free CDN) and triggered ∼50-fold higher IRF3 activation. In multiple syngeneic tumour models, a single low dose (0.5–1.25 µg) of CDN-conjugated nanoparticles, in combination with checkpoint blockade, resulted in 60–100% tumour rejection and robust immune memory upon rechallenge. Notably, while the spleen was not required for initial tumour clearance, it was essential for memory formation, as splenectomised mice failed to reject tumours upon rechallenge. These findings emphasise how PBAE structure and linker chemistry can be rationally designed to support not only immune activation but also durable immunological memory, positioning PBAEs as dynamic immunotherapeutic agents rather than passive carriers.

In another study for the targeted PBAE complexes pioneered by Stephan and co-workers,^154^ the authors showed the utility of this delivery system to produce transient engineered T cells *in situ* using direct administration compared to traditional *ex vivo* manipulation. Similar to the above studies, PGA coated PBAE complexes were produced, this time conjugating an anti-CD8 antibody to target these cells. Importantly they observed that the T-cell targeted delivery system was able to efficiently deliver and express mRNA encoding for a leukemia-specific 1928z CAR (75% of cells) and a high affinity hepatitis B virus-specific T-cell receptor (89% of cells) for chronic hepatitis B infection. *In vivo* studies in a leukaemia murine model showed that a multidose therapy using the 1928z CAR mRNA loaded PBAE complexes, administered systemically, was able to eradicate disease in 6 out of 10 mice, with the others showing some form of tumour regression. Flow cytometry analysis showed that 10% of all circulating CD8^+^ T-cells were CAR^+^ 24 h after administration, reducing to 0.8% on day 7, showcasing the transient nature of this manipulation. Interestingly the authors found that their targeted PBAE complexes displayed substantially poorer response when using a prostate tumour specific CAR mRNA construct, with increased infiltration of T-cells to the tumour but limited disease regression. This was attributed to further mutations and selection pressure that the CARs create, with further analysis of the tumours showing a reduction in target antigen expression and has been reported clinical as a challenge when inducing single antigen targeting T-cells for heterogenous cancers. Finally, this targeted delivery system was also shown to be effective for hepatitis B virus, with TCR expressing T-cells produced *in vivo* from a HBcore18-27 mRNA construct, able to regress hepatic disease 13-fold compared to the control. Together, these studies highlight the modularity and adaptability of PBAEs, whether achieved through direct functionalization, conjugation, or co-formulation approaches. These examples, including PBAE-conjugated systems, are summarized in Table 2, which outlines the type of functionalization, target immune cell, cargo, application, and reported outcomes.

**Table 2:** Summary of PBAE-based polymer functionalization and immune-targeted delivery strategies. Examples include direct chemical conjugation (*e.g.*, mannose, lentinan, antibodies) and conjugation with immune-stimulatory molecules (*e.g.*, STING agonists). Reported outcomes demonstrate enhanced immune targeting, transfection efficiency, and immunomodulatory efficacy across applications ranging from vaccines to adoptive cell therapies

Functionalization strategy	Target	Application	Reported outcomes	Reference
Mannose end-group modification *via* allyl-α-d-mannopyranoside conjugation	Dendritic cells, macrophages (APCs)	Vaccine delivery; humoral immune response induction	↑ Uptake & transfection in RAW264.7 cells; robust IgG titers; adjuvant-free immunization	Pfeifer *et al.*^97^
PLGA/PBAE co-formulation displaying MHC proteins and anti-CD28 (aAPCs)	CD8^+^ and CD4^+^ T cells	Artificial APCs for T-cell activation	↑ T-cell expansion & activation; tumour growth reduction; improved survival in melanoma model	Green *et al.*^101^
Lentinan-functionalized PBAE-G nanodiamonds (LNT-PBAE-G-ND)	Macrophages	mRNA vaccine; macrophage activation	↑ Cytokine secretion (IL-1β, IL-6, IL-12, TNF-α); strong humoral & cellular immunity; activation of cGAS–STING pathway	Wang *et al.*^177^
PBAE–STING agonist (CDN) conjugates	Systemic immune cells; APCs	Immunotherapy; checkpoint blockade combination	↑ IRF3 activation (∼50-fold); 60–100% tumour rejection; durable immune memory	Artzi *et al.*^178^
Anti-CD8 antibody–conjugated PBAE complexes	CD8^+^ T cells	*In situ* T-cell engineering; cancer & viral therapy	↑ mRNA delivery (up to 89% expression); tumour eradication in 6/10 mice; transient CAR expression; effective HBV regression	Stephan *et al.*^154^

## Discussion and critical insights

The clinical importance of PBAEs lies in their versatile properties and potential applications in various biomedical fields, particularly in immunotherapy, immune-oncology, and vaccination. PBAEs have attracted significant interest due to their ability to serve as effective delivery systems for a wide range of therapeutics, including nucleic acids, small molecules, and cells, targeting immune-related disorders and diseases. In this discussion, we will explore the clinical importance of PBAEs by highlighting their key attributes and potential clinical applications.

PBAEs offer unique advantages as delivery vectors, including pH-dependent degradation and the ability to form nanoparticles with negatively charged molecules. These properties enable efficient encapsulation and targeted delivery of therapeutics to immune cells and tissues, enhancing their bioavailability and efficacy. This enhanced delivery capability is particularly advantageous in the context of immunotherapy, where precise targeting of immune cells is crucial for modulating immune responses and treating immune-related disorders. In addition, PBAEs can be tailored to incorporate specific functionalities that facilitate immunomodulation and targeted delivery to immune cells. By engineering PBAEs with immune-targeting ligands or immunostimulatory moieties, researchers can design delivery systems capable of precisely modulating immune cell function and enhancing therapeutic outcomes. This ability to target selectively immune cells holds immense potential for developing targeted immunotherapies and vaccines for various immune-related diseases, including cancer, autoimmune disorders, and infectious diseases.

Furthermore, PBAEs have emerged as promising platforms for vaccine delivery, offering a versatile and customizable approach to enhance vaccine efficacy and immunogenicity. By encapsulating antigens or nucleic acid vaccines within PBAE nanoparticles, it is possible to improve antigen stability, promote antigen uptake by antigen-presenting cells, and enhance the induction of robust immune responses. This has significant implications for the development of next-generation vaccines against infectious diseases and cancer, where traditional vaccine approaches may be limited by antigenicity or immunogenicity issues.

Finally, the clinical translation of PBAE-based delivery systems holds promise for addressing unmet medical needs and improving patient outcomes in immunotherapy and vaccination. Several preclinical studies have demonstrated the therapeutic potential of PBAE-based formulations in various disease models, highlighting their efficacy in enhancing immune responses, suppressing tumour growth, and mitigating autoimmune pathology. As these promising preclinical results continue to advance towards clinical evaluation, PBAEs have the potential to revolutionize the treatment landscape for immune-related disorders and diseases, offering safe, effective, and targeted therapeutic interventions. In conclusion, PBAEs represent a versatile platform for the delivery of therapeutics in immunotherapy, immune-oncology, and vaccination. Their unique properties enable efficient encapsulation and targeted delivery of therapeutics to immune cells and tissues, offering opportunities for precise immunomodulation and enhanced therapeutic outcomes. With ongoing advancements in PBAE synthesis, formulation, and clinical translation, PBAEs may yet be used to address unmet medical needs and improving patient outcomes in immune-related applications.

## Comparison with clinically validated delivery platforms

Over the last decade, significant advances have been made not only in new medicinal technologies, but also in the regulatory and manufacturing framework of novel vaccines and immunotherapies. We have now seen the first clinical use of mRNA and DNA vaccines, genetically engineered cell therapies for cancer, and approval of oligonucleotides and gene editing technologies for several inherited disorders. These represent a step change away from conventional chemotherapeutics to treat disease. Although there have been marked advancements in these therapeutic modalities, there has been little clinical advance for PBAEs since their first use in the biomedical field by Langer and co-workers in 2000 for both immunological and non-immunological applications.^130^ During this same period, LNP technology has been approved for three nucleic acid medicines (Onpattro, SpikeVax, Comirnaty) whilst multiple other lipids containing formulations are in clinical use for formulation of vaccines and chemotherapeutics.

Although the scientific literature is extremely rich, with hundreds of papers displaying the promise of PBAEs, with excellent potency and tolerability in cell culture and small animal studies, to our knowledge this is yet to translate to a single clinical trial. There is of course background IP (Intellectual property) associated with PBAEs, summarised in Table 3, ranging from PBAE chemistry, use cases for pulmonary administration and co-formulation with other excipients. Further examples also include IP focusing on nucleic acid technologies and ligand functionalised nanoparticles to reprogram immune cells. It is of course plausible that established platforms such as LNPs demonstrate significantly lower risk for pharmaceutical companies. The inherent molar mass dispersity of PBAEs also may pose unpredictable pharmacokinetics and pharmacodynamics, hence the preference for assembled discrete lipids given the large body of evidence regarding LNPs safety profiles, despite the substantially easier synthetic route for PBAEs. Yet, as nucleic acid therapeutic and vaccines continue their expansion into the biomedical technology field, where PBAEs excel the most, the commercial factors relating to licensing fees for high performing ionisable lipids may shift attention towards alternative delivery systems, such as PBAEs, particularly for spin-outs and small to medium size enterprises.

**Table 3:** Background IP associated with PBAEs

Patent number	Assigned to	Chemical composition and formulation claims	Applications	Application granted date
US8287849B2	Massachusetts Institute of Technology	• Utilization of bis(acrylate ester) in the synthesis process	• Encapsulation of therapeutic, diagnostic, and/or prophylactic agents, including polynucleotides, to form nanoscale polyplexes and microparticles	16/10/2012
		• Incorporation of either bis(secondary amines) or primary amines in the polymerisation reaction		
US8562966B2	National Institutes of Health (NIH), U.S. Dept. of Health and Human Services (DHHS), U.S. Government	• Introduction and modification of various end groups of poly(β-amino esters) to yield specific properties	• Encapsulation of therapeutic, diagnostic, and/or prophylactic agents, including polynucleotides, to form nanoscale polyplexes and microparticles	22/10/2013
			• Application in tissue engineering, and biomaterials	
US20190125874A1	Massachusetts Institute of Technology	• Production of poly(β-amino esters) with branched structures	• Nebulization for non-viral delivery of therapeutic agents, especially mRNA, to lung cells	11/10/2022
			• Focus on mRNA delivery to lung cells	
US11136597B2	Yale University, Carnegie Mellon University	• Preferred use of poly(lactic-*co*-glycolic acid) (PLGA) alone or in combination with PBAEs in the nanoparticles with a PBAE content ranging from about 10 to about 20 percent (wt%) in the nanoparticles	• Utilization of advanced gene editing techniques, such as triplex-forming oligonucleotides, CRISPR, zinc finger nucleases, and TALENS, combined with gene modification potentiating agents like stem cell factor (SCF), CHK1 or ATR inhibitors, or a combination thereof. Nanoparticle are developed for efficient intracellular delivery of the gene editing components, making this approach particularly advantageous for *in vivo* applications	05/10/2021
US20210252154A1	Johns Hopkins University	• Creation of cationic polymers with diverse substituents distributed throughout the backbone	• Encapsulation of therapeutic, diagnostic, and/or prophylactic agents, including polynucleotides, to form nanoscale polyplexes and microparticles	Not yet issued
US20210220287A1	Johns Hopkins University	• Construction of multilayer particles consisting of a core and one or more layers. Core materials may include compounds of PBAE, gold nanoparticles, inorganic nanoparticles, or organic polymers	• Utilization of polymeric nanoparticles, microparticles, and gels for delivering peptide cargo	Not yet issued
		• Layer materials may include compounds of PBAE, organic polymers, peptides, and additional biological agents	• Treatment of diseases, including angiogenesis-dependent diseases	
		• Additional aspects include the provision of microparticles containing compounds of PBAE, poly(lactide-*co*-glycolide) (PLGA), or combinations	• Specific mention of age-related macular degeneration and cancer as examples	
US8808681B2	Massachusetts Institute of Technology	• Formation of crosslinked, acrylate-terminated PBAEs using various crosslinking agents	• Particularly beneficial as drug delivery vehicles and tissue engineering scaffolds	19/08/2014
		• Crosslinking process occurs through a free radical mediated process including a thermal initiator or a photoinitiator	• Application extends to fabricating microdevices, plastics, coatings, adhesives, inks, *etc.*	

From a translational standpoint, PBAEs offer opportunities that may complement established platforms like LNPs. Compared with LNPs, PBAEs provide greater tunability in polymer chemistry and nanoparticle design, enabling immune-targeted delivery and modulation of immune cells. However, PBAEs currently face challenges in formulation stability, reproducibility, scalable manufacturing, and limited clinical data, whereas LNPs benefit from extensive clinical validation, well-characterized safety, and proven scalability. By addressing these hurdles, PBAEs could serve as a versatile alternative or adjunct to LNPs, expanding the toolkit for next-generation immunotherapies and vaccines.

## Outlook: engineering the next generation of immunoactive PBAEs

PBAEs have undoubtedly revolutionised our ability to deliver important biological active molecules, including proteins, small molecules but most commonly, nucleic acids *in vitro* and in pre-clinical models. Their facile synthetic and purification procedures have put this technology in the hands of many non-chemists and opened the possibility of high throughput multifactorial libraries to produce hundreds of unique polymers in a material and time economic manner. As a consequence, PBAEs have been widely used in immunology related applications, which have been summarised and critically evaluated in this review. Given their potency in protecting and delivering nucleic acids, it is likely that future clinical translation will come from mRNA or DNA vaccines and therapeutics or therapeutic oligonucleotides as discussed above. Furthermore, the large datasets emerging from the many high throughput screens will certainly be exploited for machine learning based predictive tools and in-depth correlative analysis to predict the performance of novel unseen PBAEs. A key focus is likely to be on structures which can intrinsically target immune cells, with early work in these areas now emerging. It is also becoming clear that the adjuvant properties of other delivery systems such as LNPs are critical to the function of the existing nucleic acid vaccines and immunotherapies, hence understanding how PBAEs can activate or suppress certain immune mechanisms will also be important for their translation. The chemistry itself of PBAEs has not been a limitation in terms of design space, however the step-growth polymerisation itself, although simple, yields a broad molar mass distribution which may be challenging for predicting the ADMET properties of these systems. Therefore, future research could also entail new synthetic methodologies for PBAEs, some of which already exist, including radical based ring-opening polymerisations.^179^ Overall, the merger of immunology and polymer science presents exciting opportunities for the development of the next-generation immunotherapies and vaccines. Continued interdisciplinary research efforts both in technology development, but also in regulation and manufacturing will be essential in unlocking the full potential of PBAEs in immunology applications, ultimately translating into improved clinical outcomes for patients.

## Author contributions

Conceptualization, H. B., R. J. K., A. E. and P. G.; writing original draft preparation, H. B., R. J. K., A. E. and P. G.; writing review and editing, H. B., R. J. K., A. E., P. G. and C. A.; supervision, C. A. and P. G.; project administration, H. B. and C. A.; funding acquisition, H. B. and C. A.; all authors have read and agreed to the published version of the manuscript.

## Conflicts of interest

All authors declare that they have no conflicts of interest.

## Data Availability

No primary research results, software or code have been included and no new data were generated or analysed as part of this review.
